# In Vitro and In Vivo Evaluation of the Effects of Drug 2c and Derivatives on Ovarian Cancer Cells

**DOI:** 10.3390/pharmaceutics16050664

**Published:** 2024-05-15

**Authors:** Marianna Maddaloni, Rossella Farra, Barbara Dapas, Fulvia Felluga, Fabio Benedetti, Federico Berti, Sara Drioli, Mattia Vidali, Maja Cemazar, Urska Kamensek, Claudio Brancolini, Erminio Murano, Francesca Maremonti, Mario Grassi, Alice Biasin, Flavio Rizzolio, Enrico Cavarzerani, Bruna Scaggiante, Roberta Bulla, Andrea Balduit, Giuseppe Ricci, Gabriella Zito, Federico Romano, Serena Bonin, Eros Azzalini, Gabriele Baj, Domenico Tierno, Gabriele Grassi

**Affiliations:** 1 Department of Life Sciences, Cattinara University Hospital, Trieste University, Strada di Fiume 447, 34149 Trieste, Italy; marianna.maddaloni@klinik.uni-regensburg.de (M.M.); rossellafarra77@gmail.com (R.F.); b.dapas@alice.it (B.D.); francesca.maremonti@uniklinikum-dresden.de (F.M.); bscaggiante@units.it (B.S.); 2 Department of Chemical and Pharmaceutical Sciences (DSCF), University of Trieste, 34127 Trieste, Italy; ffelluga@units.it (F.F.); benedett@units.it (F.B.); fberti@units.it (F.B.); sdrioli@units.it (S.D.); mattia.vidali@outlook.com (M.V.); 3 Department of Experimental Oncology, Institute of Oncology Ljubljana, Zaloska 2, SI-1000 Ljubljana, Slovenia; mcemazar@onko-i.si (M.C.); ukamensek@onko-i.si (U.K.); 4 Faculty of Health Sciences, University of Primorska, Polje 42, SI-6310 Izola, Slovenia; 5 Laboratory of Epigenomics, Department of Medicine, University of Udine, Piazzale Kolbe 4, 33100 Udine, Italy; claudio.brancolini@uniud.it; 6 Nealys S.R.L., Via Flavia 23/1, 34148 Trieste, Italy; murano.erminio@libero.it; 7 Department of Engineering and Architecture, University of Trieste, Via Valerio 6/A, 34127 Trieste, Italy; mario.grassi@dia.units.it (M.G.); alice.biasin@phd.units.it (A.B.); 8 Pathology Unit, Centro di Riferimento Oncologico di Aviano (CRO) IRCCS, 33081 Aviano, Italy; flavio.rizzolio@unive.it; 9 Department of Molecular Sciences and Nanosystems, Ca’ Foscari University of Venice, 30172 Venice, Italy; enrico.cavarzerani@gmail.com; 10 Department of Life Sciences, University of Trieste, 34127 Trieste, Italy; rbulla@units.it (R.B.); gbaj@units.it (G.B.); 11 Institute for Maternal and Child Health, IRCCS Burlo Garofolo, 34137 Trieste, Italy; abalduit@units.it (A.B.); giuseppe.ricci@burlo.trieste.it (G.R.); gabriella.zito@burlo.trieste.it (G.Z.); federico.romano@burlo.trieste.it (F.R.); 12 Department of Medical, Surgical and Health Science, University of Trieste, 34129 Trieste, Italy; sbonin@units.it (S.B.); eazzalini@units.it (E.A.)

**Keywords:** ovarian cancer, 2C, apoptosis, E2F1, in silico docking

## Abstract

Background: The identification of novel therapeutic strategies for ovarian cancer (OC), the most lethal gynecological neoplasm, is of utmost urgency. Here, we have tested the effectiveness of the compound 2c (4-hydroxy-2,6-bis(4-nitrobenzylidene)cyclohexanone 2). 2c interferes with the cysteine-dependent deubiquitinating enzyme (DUB) UCHL5, thus affecting the ubiquitin-proteasome-dependent degradation of proteins. Methods: 2c phenotypic/molecular effects were studied in two OC 2D/3D culture models and in a mouse xenograft model. Furthermore, we propose an in silico model of 2c interaction with DUB-UCHL5. Finally, we have tested the effect of 2c conjugated to several linkers to generate 2c/derivatives usable for improved drug delivery. Results: 2c effectively impairs the OC cell line and primary tumor cell viability in both 2D and 3D conditions. The effectiveness is confirmed in a xenograft mouse model of OC. We show that 2c impairs proteasome activity and triggers apoptosis, most likely by interacting with DUB-UCHL5. We also propose a mechanism for the interaction with DUB-UCHL5 via an in silico evaluation of the enzyme-inhibitor complex. 2c also reduces cell growth by down-regulating the level of the transcription factor E2F1. Eventually, 2c activity is often retained after the conjugation with linkers. Conclusion: Our data strongly support the potential therapeutic value of 2c/derivatives in OC.

## 1. Introduction

Being the seventh most common cancer and the fifth cause of cancer death among females, ovarian cancer (OC) is the most lethal gynecological neoplasm [1]. An aggressive front-line platinum-based chemotherapy represents the basis for the treatment of OC. While resulting in about 80% of the curative rate, this approach frequently leads to tumor chemo-resistance, with the consequent disease recurrence within 18–24 months and a five-year survival rate below 45% [2,3]. Thus, partial improvements in terms of patient survival have been achieved in the last few years.

From the histological point of view, epithelial ovarian cancers (EOCs) are the most common forms of OCs. EOCs represent a heterogeneous group of tumors with different epidemiology, prognosis, and molecular profiles [4]. High-grade serous carcinoma (HGSOC, 70% of all EOC cases), low-grade serous carcinoma (LGSOC, <5% of all EOCs), endometrioid carcinoma (10%), clear cell carcinoma (10%), and mucinous carcinoma (3%) are the most common subtypes of EOCs [5]. HGSOC is the most aggressive histotype and exhibits a marked tendency to extra-ovarian dissemination; less than 35% of patients with advanced HGSOC survive longer than 5 years after diagnosis. 

**Figure 1:**
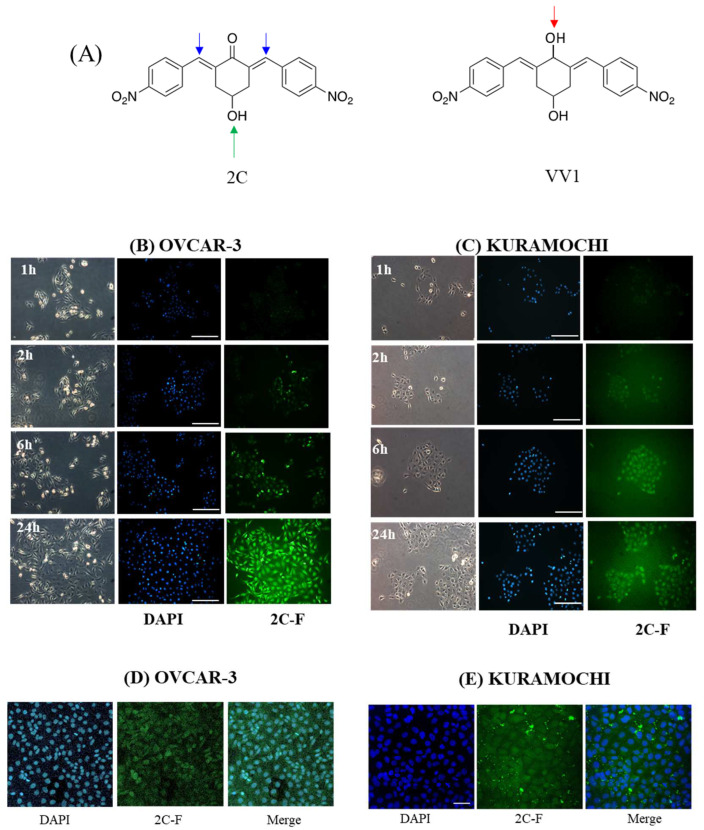
2c Uptake in OVCAR3 and Kuramochi cells. (**A**) 2c is a 4-hydroxy-2,6-bis(4-nitrobenzylidene)cyclohexanone; the presence of the hydroxy group (green arrow) allows for further modifications on the 4-hydroxycyclohexanone scaffold; blue arrows indicate sites able to bind to cysteine residues present in deubiquitinase enzymes. VV1 differs from 2c for the presence of an OH group (red arrow), which makes the molecule inactive. (**B**,**C**) representative images of OVCAR3 and Kuramochi, respectively, treated by 2c (2 μM) fluorescently labelled (2c-F); images were taken at different time intervals; scale bar: 100 μm, magnification 20×; nuclei have been labelled by DAPI (blue); images were taken by a Leica DM2000 fluorescence microscope. (**D**,**E**) representative confocal images of OVCAR3 and Kuramochi, respectively, treated by 2c (2 μM) fluorescently labelled (2c-F); images were taken 24 h after drug administration by a Nikon Eclipse C1si confocal microscope; nuclei have been labelled by DAPI (blue); scale bar: 50 μm, magnification 20×.

Based on the above considerations, it is clear that the identification of novel therapeutic strategies for OC is extremely urgent. We showed that 4-Hydroxy-2,6-bis(4-nitrobenzylidene)cyclohexanone 2 (2c, Figure 1A) has a broad spectrum of anti-tumor activity at the μM level against different tumor cell lines [6]. Moreover, we showed that it inhibits several cysteine-dependent deubiquitinating enzymes (DUBs). Some DUBs belong to the ubiquitin–proteasome system (UPS), which disposes cellular proteins to be eliminated via the conjugation of ubiquitin molecules, particularly when K48 chains are generated [7]. Once poly-ubiquitinylated, proteins become the substrate for a protein complex (proteasome), which degrades them [8]. This process is essential for controlling many vital cellular processes, especially in cancer cells, which tend to accumulate altered proteins that must be rapidly degraded. Thus, UPS impairment causes the accumulation of polyubiquitinated proteins, leading to proteotoxic stress and cell apoptosis [6,9]. While cysteine-dependent DUBs are not the unique target of 2c, inhibition of the UPS is likely the major component of the observed antitumor activity [10]. Recently, we have shown that 2c can affect the viability of a panel of OC cell lines and that its effectiveness relates to the intra-mechanical features of the specific cell phenotype [11]. Here, we deepen the study about 2c effectiveness by exploring the phenotypic/molecular mechanisms ruling 2c effects in OC cell lines. We also extend the investigation to patient-derived cancer cells (cultured in 2D/3D models) and to a xenograft mouse model of OC. We also provide in silico evidence of the possible interaction mechanism between 2c and DUB-UCHL5. Finally, we test the effectiveness of several novel 2c derivatives to allow future conjugation with smart moieties for improved drug delivery/targeting/specificity. Together, our results support the potential therapeutic value of 2c and its derivatives in OC.

## 2. Materials and Methods

### 2.1. Cell Culture

Two human ovarian cancer cell lines, OVCAR3 and Kuramochi, were used [12]. Both cell lines were cultured in RPMI-1640 (EuroClone^®^, Pero, Milano, Italy) integrated with 10% heat-inactivated fetal bovine serum (Gibco™ by Life Technologies, Thermo Fisher Scientific, Waltham, MA, USA), 100 U/mL of penicillin and 100 μg/mL of streptomycin (EuroClone^®^), 2 mM L-glutamine (EuroClone^®^): Culture medium for OVCAR3 was also integrated with 0.01 mg/mL bovine insulin (Sigma-Aldrich^®^, St. Louis, MA, USA). All cell lines were grown at 37 °C in a humidified atmosphere at 5% CO_2_. 

Patient-derived OC cells were isolated from peritoneal fluids of patients enrolled at the Obstetrics and Gynecology Department of the Institute for Maternal and Child Health IRCCS “Burlo Garofolo” of Trieste (Italy), who underwent laparoscopy or laparotomy after diagnosis of a pelvic mass. All patients agreed to sign an informed consent form, following approval of the ethical considerations by the Comitato Etico Unico Regionale (CEUR, protocol number 4829), the regional ethical committee for Friuli Venezia-Giulia, Italy. Peritoneal fluids were filtered through a 300 µm cell strainer (PluriSelect) to remove macroscopic debris and centrifuged at 250× *g* for 7 min. Red blood cells (RBC) were removed by RBC Lysis Buffer (Roche). Cells were then resuspended and cultured in Human Endothelial Serum Free Medium (Gibco, Thermo Fisher Scientific) supplemented with 20 ng/mL of epidermal growth factor (Società Italiana Chimici, Life Sciences, Roma, Italy), 10 ng/mL basic fibroblast growth factor (Società Italiana Chimici, Life Sciences), 1% penicillin-streptomycin (Sigma-Aldrich), and 10% heat-inactivated fetal bovine serum (Gibco, Thermo Fisher Scientific). Cells were maintained at 37 °C in a 5% *v*/*v* CO_2_ incubator in a humidified atmosphere. The medium was changed every 2–3 days. To determine the purity of patient-derived OC cells, cytofluorimetric analysis and immunofluorescence assays were performed using the following markers: Epithelial membrane antigen (EMA)/mucin-1, vimentin, cytokeratin 8/18, CD44, and Wilm’s tumor 1 (WT1), as previously described [13]. In addition, CD45 and von Willebrand factor were tested to exclude leukocyte and endothelial cell contamination, respectively.

### 2.2. Uptake Studies

Due to the hydrophobic nature of 2c and its derivatives, the compounds tested were resuspended in DMSO. 7.2 × 10^4^ OVCAR3 or Kuramochi/well were seeded on glass slides in 6-well plates; 24 h after seeding, cells were treated with 2 μM of 2c labelled by fluorophore 4-N-propylamino-1,8-naphthalimide (2c-F) or fluorescein (VV1-Fl, compounds 10-Fl and 11-Fl). At different time intervals, cells were fixed in 4% paraformaldehyde (Sigma-Aldrich) and stained with DAPI; images were then taken by a Leica DM2000 fluorescence microscope (20 × objective). Confocal images were taken with the Nikon Eclipse C1 microscope, following the same preparation procedure described above.

### 2.3. Drug Synthesis

NMR spectra were recorded on a Varian 500 MHz spectrometer in the indicated solvents at 500 MHz (^1^H) and 125 MHz (^13^C) or on a Varian 400 at 400 MHz (^1^H) and 100 MHz (^13^C); chemical shifts are in ppm (δ) in the specified solvents. Coupling constants J are given in Hertz. ^1^H and ^13^C NMR resonances were assigned using a combination of DEPT, COSY, and HSQC spectra. Electrospray (ESI) mass spectra were obtained on a Bruker Daltonics Esquire 4000 spectrometer. High-resolution mass spectra were obtained on a Bruker micrOTOF-Q. Flash chromatography was performed on silica gel 60 (Merck, 230–400 mesh). Yields refer to spectroscopically (^1^H NMR) homogeneous materials. Commercial reagents and solvents were purchased from Sigma-Aldrich. 2c (**1**), 2c-OSu (**1a**), 1-[(tert-butoxycarbonyl)amino]-5-aminopentane, L-Phenylalanylamide ((S)-2-Amino-3-phenylpropanamide) [8], 1-[(tert-Butoxycarbonyl)amino]-3-aminopropane, and L-leucineamide ((2S)-2-amino-4-methylpentanamide) were prepared by the indicated literature procedures [6,10,14,15]. N-Boc deprotection was carried out in a 10% TFA solution in DCM, followed by solvent evaporation. Flash chromatography (FC) was run with CHCl_3_ as the eluent. Yields refer to purified products.

Synthesis of VV1 (4-hydroxy-2,6-bis(4-nitrobenzylidene)cyclohexanol). To a solution of 2c (**1**) (0.152 g, 0.4 mmol) in a 1:9 MeOH/THF solution (25 mL), NaBH_4_ (0.015 g, 0.40 mmol) was added at 0 °C under stirring. After 4 h at rt, brine (50 mL) was added, and the resulting solution was extracted with diethyl ether. Evaporation of the solvent gave VV1 as a 3:2 mixture of cis/trans diastereoisomers. Yellowish solid, 93% yield. NMR spectra are given for the diastereoisomeric mixture. ^1^H NMR (400 MHz, DMSO-d_6_), δ 8.19, 8.17 (2d, ratio 3:2, 4H, o-NO_2_Ph*H*), 7.57, 7.53 (2d, ratio 2:3, 4H, m-NO_2_Ph*H*), 6.76, 6.74 (2s, ratio 2:3, 2H, 2 × C*H* = C), 5.82, 5.74 (2d, ratio 3:2, 1H, J = 4.7 Hz each, C(1)-O*H*), 5.05, 4.99 (2d, ratio 3:2, 1H, J = 4.0 Hz each, C(4)-O*H*), 4.74, 4.67 (2 bm, ratio 3:2, 1H, H-1), 3.87, 3.65 (2m, ratio 3:2, 1H, H-4), 2.95, 2.33 (2 dd, J = 13.7, 9.3 and 3.8 Hz, major diastereoisomer ring CH_2_), 2.73, 2.55 (dd, J = 13.3, 6.5, 3.3 Hz, minor diastereoisomer, ring CH_2_). ^13^C NMR (100 MHz, DMSO-d_6_), δ 146.08, 145.48, 145.09, 145.01, 144.59, 130.28, 130.25, 123.91, 123.85, 121.87, 120.83, 76.56, 75.49, 68.71, 68.14, 36.34, 35.18. ESI-MS, *m*/*z*: 383.1 [M + H]^+^. HRMS, *m*/*z* (negative ion) found: 381.1090 [M − H]^−^; Calcd. for C_20_H_17_N_2_O_6_: 381.1092.

Due to the hydrophobic nature of 2c and VV1, the two compounds were administered diluted in DMSO (dimethyl sulfoxide).

*Coupling of 2c-OSu with amines* (Supplementary Materials Figure S5). General procedure for the synthesis of compounds **2**–**12**. To a solution of 2c-OSu (**1a**) [(3E, 5E)-3,5-bis[(4-nitrophenyl)methylidene]-4-oxocyclohexyl 2,5-dioxo-1-pyrrolidine-1-carboxylate] (0.520 g, 1.0 mmol) in anhydrous DCM, the amine partner (1.2 mmol) was added. The reaction mixture was stirred at 25 °C for 18 h, then was washed with 1N aq HCl or 5% aq. citric acid and brine. Evaporation of the solvent left a residue that was purified as indicated.
*[(3E, 5E)-3,5-bis[(4-nitrophenyl)methylidene]-4-oxocyclohexyl N-propylcarbamate (***2***).* From **1a** and n-propylamine, yellow solid, 75% yield after FC; ^1^H NMR (500 MHz, DMSO-d_6_), δ 8.28 (app. d, J = 8.7 Hz 4H, 2 × o-NO_2_Ph*H*), 7.79 (s, 2H, 2 × C*H* = C), 7.78 (app. d, J = 8.7 Hz, 2 × m-NO_2_Ph*H*), 7.05 (t, J = 5.6 Hz, 1H, CON*H*), 5.01 (s, 1H, C*H*OCO), 3.19 (m, 4H, 2 × ring CH_2_), 2.76 (app q, J = 7.4 Hz, 2H, C*H*_2_NH), 1.22 (sext, J = 7.4 Hz, 2H, CH_2_C*H*_2_CH_3_), 0.66 (t, J = 7.4 Hz, 3H, CH_2_CH_2_C*H*_3_) ppm; ^13^C NMR (125 MHz, DMSO-d_6_), δ 187.75, 155.65, 147.48, 141.99, 136.19, 135.95, 131.69, 124.07, 66.85, 42.26, 32.94, 22.88, 11.50 ppm. ESI-MS, *m*/*z*: 488.1 [M + Na]^+^.
*[(3E, 5E)-3,5-bis[(4-nitrophenyl)methylidene]-4-oxocyclohexyl N-[2-(2-hydroxyethoxy)ethyl]carbamate (***3***).* From **2a** and 2-(2-aminoethoxy)ethanol, light yellow solid, 65% yield after FC; ^1^H NMR (400 MHz, CDCl_3_), δ 8.29 (app. d, J = 8.7 Hz, 4H, 2 × o-NO_2_Ph*H*), 7.91 (s, 2H, 2 × C*H* = C), 7.60 (4H, app. d, J = 8.7 Hz, 2 × m-NO_2_Ph*H*), 5.17 (br, 1H, C*H*OCO), 5.03 (br, 1H, NH), 3.71 (m, 2H, C*H_2_*OH), 3.49 (2H, NHCH_2_C*H*_2_O), 3.52 (t, 2H, OC*H*_2_CH_2_OH), 3.30 (m, 2H, C*H*_2_NH), 3.26–3.13 (m, 4H, 2 × ring CH_2_) ppm; ^13^C NMR (100 MHz, CDCl_3_), δ 187.56, 155.38, 147.48, 141.54, 137.15, 134.43, 130.73, 123.77, 72.15, 69.86, 67.06, 61.60, 40.73, 33.18 ppm. ESI-MS, *m*/*z*: 534.2 [M + Na]^+^. HRMS, *m*/*z*, found: 535.1484; Calcd for [C_25_H_25_N_3_O_9_Na]^+^ 535.1483.
*[(3E, 5E)-3,5-bis[(4-nitrophenyl)methylidene]-4-oxocyclohexyl N-[(naphthalen-2-yl)methyl]carbamate (***4***).* From **1a** and 2-naphtylmethylamine, yellow-orange solid, 56% yield after FC; ^1^H NMR (400 MHz, DMSO-d_6_), δ 8.26 (d, J = 8.5 Hz, 4H, 2 × o-NO_2_Ph*H*), 7.82-7.80 (m, 8H, 2 × C*H* = C, 2 × m-NO_2_Ph*H*, N*H*COO, 1 Ar*H*), 7.72 (d, J = 8.3 Hz, 1H, Ar*H*), 7.68 (m, 4H, Ar*H*), 7.24 (d, J = 8.3 Hz, 1H, Ar*H*), 5.09 (m, 1H, C*H*OCO), 4.19 (d, J = 5.9 Hz, 2H, NHC*H*_2_Ar), 3.25 (m, 4H, 2 × ring CH_2_) ppm; ^13^C NMR (100 MHz, DMSO-d_6_), δ 187.63, 155.94, 147.43, 141.94, 137.57, 136.26, 135.83, 133.12, 132.40, 131.66, 128.18, 127.85, 127.80, 126.48, 126.01, 125.89, 125.34, 124.04, 67.22, 44.18, 32.92 ppm; ESI-MS: 586.2 *m*/*z* [M + Na]^+^;
*[(3E, 5E)-3,5-bis[(4-nitrophenyl)methylidene]-4-oxocyclohexyl N-[(naphthalen-1-yl)methyl]carbamate (***5***).* From **1a** and 1-naphtylmethylamine, orange-red solid, 82% yield after FC; ^1^H NMR (500 MHz, DMSO-d_6_), δ 8.26 (app d, J = 8.5 Hz, 4H, 2 × o-NO_2_Ph*H*), 7.96 (d, J = 8.5 Hz, 1H, Ar*H*), 7.87 (d, J = 8.5 Hz, 1H, Ar*H*), 7.81-7.73 (m, 5H, 2 × C*H* = C, N*H*COO, 2 Ar*H*), 7.44 (t, 1H, Ar*H*), 7.31 (t, 1H, Ar*H*), 7.21 (d, J = 7.0 Hz, 1H, Ar*H*), 5.10 (bs, 1H, C*H*OCO), 4.49 (d, J = 5.8 Hz, 2H, ArC*H*_2_NH), 3.25 (m, 4H, 2 × ring CH_2_) ppm; ^13^C NMR (125 MHz, DMSO-d_6_), δ 187.63, 155.78, 147.46, 141.96, 136.26, 135.83, 135.05, 133.60, 131.67, 131.04, 128.87, 127.82, 126.45, 126.09, 125.65, 125.28, 124.06, 123.66, 66.22, 42.08, 32.91 ppm; ESI-MS: 586.2 [M + Na]^+^.
*[(3E, 5E)-3,5-bis[(4-nitrophenyl)methylidene]-4-oxocyclohexyl N-(naphthalen-1-yl)carbamate (***6***).* From **1a** and 1-naphthylamine in the ratio 1:1.5, light yellow solid, 61% yield after FC; ^1^H NMR (400 MHz, DMSO-d_6_) δ 9.46 (bs, CON*H*-Ar), 8.29 (app.d, 4H, 2 × o-NO_2_Ph*H*), 7.88-7.80 (m, 7H, 2 × C*H* = C, 2 × m-NO_2_Ph*H,* 1 Ar*H*), 7.77 (d, J = 8.3 Hz, 1H, Ar*H*), 7.70 (d, J = 8.3 Hz, 1H, Ar*H*), 7.49–7.45 (m, 1H, Ar*H*), 7.40–7.35 (m, 2H, Ar*H*), 7.30 (d, J = 7.35 Hz, 1H, Ar*H*), 5.17 (m, 1H, C*H*OCO), 3.32 (m, 4H, 2 × ring CH_2_) ppm; ^13^C NMR (100 MHz, DMSO-d_6_) δ 187.74, 154.46, 147.49, 142.01, 136.35, 135.88, 134.06, 133.75, 131.71, 128.62, 128.40, 126.38, 126.11, 125.86, 125.79, 124.08, 123.10, 122.26, 68.04, 32.74 ppm; ESI-MS: 572.2 *m*/*z* [M + Na]^+^.
*[(3E, 5E)-3,5-bis[(4-nitrophenyl)methylidene]-4-oxocyclohexyl N-(3-aminopropyl)carbamate (trifluoroacetate salt) (***7***)*. **1a** and 1-[(tert-butoxycarbonyl)amino]-3-aminopropane [14] gave the corresponding N-Boc protected amine (**7a**); yellow solid, 82% yield after FC; ^1^H NMR (400 MHz, CDCl_3_): δ 8.29 (apparent d, J = 8.7 Hz, 4H, 2 × o-NO_2_PhH), 7.91 (s, 2H, 2 C = CH), 7.60 (app. d, J = 8.7 Hz, 4H, 2 × m-NO_2_PhH), 5.24 (br, 1H), 5.13 (br, ^1^H, CHOCO), 4.67 (br, 1H), 3.21–3.05 (m, 8H, 2 × CH_2_ ring and 2 × CH_2_NHCO), 1.55 (m, 2H, CH_2_cH_2_cH_2_), 1.42 (s, 9H, Boc) ppm; 13C NMR (100 MHz, CDCl_3_): δ 187.49, 156.42, 155.45, 147.52, 141.59, 137.19, 134.51, 130.72, 123.78, 79.48, 67.11, 37.46, 36.96, 33.26, 30.47, 28.34 ppm. N-Boc deprotection of **7a** gave quantitatively the ammonium salt **7** as a yellow solid product that was washed on the filter with cold H_2_O; 1H NMR (400 MHz, DMSO-d6): δ 8.26 (app d, J = 8.7 Hz, 4H, 2 × o-NO_2_ PhH), 7.78 (s, 2H, 2 × C = CH), 7.77 (app. d, J = 8.7 Hz, 4H, 2 × m-NO_2_ PhH), 7.52 (broad, 3H, NH_3_^+^), 7.19 (t, J = 5.6 Hz, 1H, CONH), 5.02 (bs, 1H, CHOCO), 3.30–3.10 (m, 4H, 2 × CH_2_ ring), 2.87 (m, 2H, CH2NHCO), 2.48 (m, 2H, CH_2_NH_3_^+^), 1.51 (quint., J = 7.9 Hz, 2H, CH_2_cH_2_cH_2_) ppm; ^13^C NMR (100 MHz, DMSO-d6): 187.55, 155.76, 147.45, 141.92, 135.75, 131.65, 124.04, 67.09, 37.70, 37.05, 32.97, 27.88 ppm; ESI-MS, *m*/*z*: 481.2 [M + H]^+^.
*[(3E, 5E)-3,5-bis[(4-nitrophenyl)methylidene]-4-oxocyclohexyl N-(3-aminopropyl)carbamate (trifluoroacetate salt) (***7***)*. **1a** and 1-[(tert-butoxycarbonyl)amino]-3-aminopropane [14] gave the corresponding N-Boc protected amine (**7a**); yellow solid, 82% yield after FC; ^1^H NMR (400 MHz, CDCl_3_), δ 8.29 (apparent d, J = 8.7 Hz, 4H, 2 × o-NO_2_Ph*H*), 7.91 (s, 2H, 2 × C*H* = C), 7.60 (app. d, J = 8.7 Hz, 4H, 2 × m-NO_2_Ph*H*), 5.24 (br, 1H, NH), 5.13 (br, 1H, C*H*OCO), 4.67 (br, 1H, NH), 3.21–3.05 (m, 8H, 2 × ring CH_2_, C*H*_2_NHBoc and C*H*_2_NHCO), 1.55 (m, 2H, CH_2_C*H*_2_CH_2_), 1.42 (s, 9H, Boc) ppm; ^13^C NMR (100 MHz, CDCl_3_), δ 187.49, 156.42, 155.45, 147.52, 141.59, 137.19, 134.51, 130.72, 123.78, 79.48, 67.11, 37.46, 36.96, 33.26, 30.47, 28.34 ppm. N-Boc deprotection of **7a** gave quantitatively the ammonium salt **7** as a yellow solid product, that was washed on the filter with cold H_2_O; ^1^H NMR (400 MHz, DMSO-d_6_), δ 8.26 (app d, J = 8.7 Hz, 4H, 2 × o-NO_2_Ph*H*), 7.78 (s, 2H, 2 × C*H* = C), 7.77 (app. d, J = 8.7 Hz, 4H, 2 × m-NO_2_ Ph*H*), 7.52 (broad, 3H, N*H*_3_^+^), 7.19 (t, J = 5.6 Hz, 1H, CON*H*), 5.02 (bs, 1H, C*H*OCO), 3.30–3.10 (m, 4H, 2 × CH_2_ ring), 2.87 (m, 2H, C*H*_2_NHCO), 2.48 (m, 2H, C*H*_2_NH_3_^+^), 1.51 (quint., J = 7.9 Hz, 2H, CH_2_C*H*_2_CH_2_) ppm; ^13^C NMR (100 MHz, DMSO-d_6_), δ 187.55, 155.76, 147.45, 141.92, 135.75, 131.65, 124.04, 67.09, 37.70, 37.05, 32.97, 27.88 ppm; ESI-MS, *m*/*z*: 481.2 [MH]^+^. HRMS *m*/*z*, found: 481.1718; Calcd. for [C_24_H_25_N_4_O_7_]^+^: 481.1718.
*[(3E, 5E)-3,5-bis[(4-nitrophenyl)methylidene]-4-oxocyclohexyl N-(5-aminopentyl) carbamate (trifluoroacetate salt) (***8***)*. **1a** and 1-[(tert-butoxycarbonyl)amino]-5-aminopentane [10] gave the corresponding N-Boc protected amine (**8a**) as a yellow solid, 86% yield after FC; ^1^H NMR (400 MHz, CDCl_3_), δ 8.27 (app d, J = 8.7 Hz, 4H, 2 × o-NO_2_Ph*H*), 7.88 (s, 2H, 2 × C*H* = C), 7.58 (app d, 4H, J = 8.7 Hz, 2 × m-NO_2_Ph*H*), 5.15 (bs, 1H, C*H*OCO), 4.72 (br, 1H, NH), 4.53 (br, 1H, NH), 3.25–3.01 (m, 8H, 2 × CH_2_ ring, C*H*_2_NHCO and C*H*_2_NHBoc), 1.43 (s, 9H, Boc), 1.33–1.18 (m, 6H, 3 × CH_2_ chain) ppm; ^13^C NMR (100 MHz, CDCl_3_), δ 187.52, 156.08, 155.25, 147.44, 141.58, 134.54, 130.71, 123.74, 78.86, 66.86, 40.71, 40.18, 33.16, 28.36, 23.67 ppm. N-Boc deprotection of **8a** gave quantitatively the ammonium salt **8** as a yellow solid product that was washed on the filter with cold H_2_O. ^1^H NMR (400 MHz, DMSO-d_6_), δ 8.28 (app. d, J = 8.7 Hz, 4H, 2 × o-NO_2_Ph*H*), 7.79 (s, 2H, 2 × C*H* = C), 7.78, (app. d, J = 8.7 Hz, 4H, 2 x m-NO_2_Ph*H*), 7.56 (broad, 3H, N*H*_3_^+^), 7.05 (t, J = 5.6 Hz 1H, CON*H*), 4.99 (bs, 1H, C*H*OCO), 3.16 (m, 4H, 2 × ring CH_2_), 2.81–2.63 (m, 4H, 2 × chain CH_2_), 1.42–1.10 (m, 6H, 3 × chain CH_2_) ppm; ^13^C NMR (100 MHz, DMSO-d_6_), δ 187.68, 171.71, 155.64, 147.49, 141.97, 136.21, 135.90, 131.70, 124.08, 66.90, 39.10, 32.98, 26.98, 23.33 ppm. ESI-MS, *m*/*z*: 509.2 [M^+^]; 363.1 [M-146; C_20_H_14_N_2_O_5_]
*3-[[(3E, 5E)-3,5-bis[(4-nitrophenyl)methylidene]-4-oxocyclohexyloxy carbonylamino]] propanoic acid (***9***).* From **1a** and β-alanine, yellow solid, triturated with EtOEt, 53% yield; ^1^H NMR (400 MHz, DMSO-d_6_), δ 12.10 (s, 1H, COO*H*), 8.28 (app d, J = 8.5 Hz, 4H, 2 × o-NO_2_Ph*H*), 7.79 (s, 2H, 2 × C*H* = C), 7.78 (app. d, J = 8.7 Hz, 4H, 2 × m-NO_2_Ph*H*), 7.11 (t, J = 5.4 Hz, 1H, N*H*CO), 5.02 (brs, 1H, C*H*OCO), 3.25-3.14 (m, 4H, 2 × CH_2_ ring), 3.01 (m, 2H, C*H*_2_NH), 2.21 (t, 2H, C*H*_2_COOH) ppm; ^13^C NMR (100 MHz, DMSO-d_6_), δ 187.72, 173.01, 155.53, 147.48, 141.98, 136.23, 135.88, 131.68, 124.09, 67.03, 55.36, 36.76, 34.29, 32.94 ppm. ESI-MS, *m*/*z*: 496.2 [M + H]^+^. HRMS, *m*/*z*, found 518.1171 [M + Na]^+^; calcd. for [C_24_H_21_N_3_O_9_Na]^+^: 518.1170.
*(2S)-2-[[(3E, 5E)-3,5-bis[(4-nitrophenyl)methylidene]-4-oxocyclohexyloxy carbonyl amino]]-3-phenylpropanoic acid (***10***).* From **1a** and L-phenylalanine, yellow solid, triturated with EtOEt, 75% yield; ^1^H NMR (500 MHz, CDCl_3_), δ 8.29–8.23 (2 app d, 4H, 2 × o-NO_2_Ph*H*), 7.90, 7.85 (2s, 2H, 2 × C*H* = C), 7.56–7.52 (m, 4H, 2 × m-NO_2_Ph*H*, J = 8.6 Hz), 7.27–7.20 (m, 5H, Ph*H*), 5.13 (br, 1H, C*H*OCO), 5.06 (d, J = 7.5 Hz, 1H, N*H*CO), 4.55 (m, 1H, C*H*(NH)CO), 3.30–3.00 (m, 4H, 2 × ring CH_2_), 3.04–2.89 (m, 2H, PhC*H*_2_) ppm; ^13^C NMR (125 MHz, CDCl_3_), δ 187.38, 175.32, 154.75, 147.55, 141.49, 137.39, 135.38, 134.20, 130.72, 129.14, 128.67, 127.27, 123.80, 67.70, 54.41, 37.43, 35.15 ppm. ESI-MS: 594.1 [M + Na]^+^.
*(2S)-6-amino-2-[[(3E, 5E)-3,5-bis[(4-nitrophenyl)methylidene]-4-oxocyclohexyloxy carbonylamino]]hexanoic acid (***11***).* From **1a** and L-lysine, yellow solid, triturated with EtOEt, 75% yield; ^1^H NMR (400 MHz, DMSO-d_6_), δ 12.46 (br, 1H, COOH), 8.29 (4H, 2 × o-NO_2_Ph*H*), 7.82–7.50 (m, 10H, 2 × C*H* = C, 2 × m-NO_2_Ph*H,* N*H*_3_^+^ and N*H*CO), 5.05 (br, 1H, C*H*OCO), 4.18–3.61 (m, 1H, Lys α-C*H*), 3.44–2.95 (m, 4H, 2 × ring CH_2_), 2.69 (br, 2H, Lys ε-C*H*_2_), 1.72–1.23 (m, 6H, Lys β-C*H*_2_, δ-C*H*_2_, γ-C*H*_2_); ^13^C NMR (100 MHz, DMSO-d_6_), δ 187.17, 173.65, 155.34, 147.02, 141.60, 135.84, 135.38, 131.15, 123.64, 66.88, 53.53, 38.48, 32.35, 29.88, 26.43, 22.45. ESI-MS: *m*/*z* 554.2 [M + H]^+^.
*[(3E, 5E)-3,5-bis[(4-nitrophenyl)methylidene]-4-oxocyclohexyl N-[(1S)-1-carbamoyl-2-phenylethyl)carbamate (***12***).* From **1a** and L-phenylalaninamide, yellow solid, 63% yield after FC; ^1^H NMR (500 MHz, CDCl_3_), δ 8.29, 8.27 (2 app d, J = 8.4 Hz each, 4H, 2 × o-NO_2_Ph*H*), 7.86 (s, 2H, C*H* = C), 7.58, (app d, 4H, J = 8.4 Hz each, m-NO_2_Ph*H*), 7.21–7.08 (m, 5H, Ph*H*), 5.36 (d, J = 6.1 Hz, N*H*COO), 5.30, 5.26 (2 br s, 1H each, CON*H*_2_), 5.14 (brs, 1H, C*H*OCO), 4.28 (app q, J = 6.9 Hz, 1H, C*H(*NH)CO), 3.25–3.10 (ddd, part AB of an ABX, J = 2.4, 6.0, 16.4 Hz, 4H, 2 × ring C*H*_2_), 3.02 (dd, J = 6.0, 13.7 Hz, part A of an ABX, PhC*H*H), 2.89 (dd, part B of an ABX, J = 7.5 and 13.7 Hz, PhCH*H*) ppm; ^13^C NMR (125 MHz, CDCl_3_), δ 187.29, 137.05, 154.78, 147.49, 141.50, 137.36, 136.10, 134.23, 130.72, 129.16, 128.67, 127.13, 123.76, 67.69, 55.68, 38.71, 33.01 ppm; ESI-MS: 593.2 [M + Na]^+^.
*[(3E, 5E)-3,5-bis[(4-nitrophenyl)methylidene]-4-oxocyclohexyl N-[3-[(2S)-2-amino-3-phenylpropanamido]propyl]carbamate (trifluoroacetate salt) (***13***)*. HOBT (0.162 g, 1.2 mmol), EDC (0.230 g, 1.2 mmol), and TEA (0.16 mL, 1.2 mmol) were added in order to yield a solution of N-Boc-(L)Phe (2.0 mmol) in the minimum amount of DCM. After 30 min stirring at 25 °C, compound **7** was added (1.0 mmol), and the apparent pH was adjusted to 9 with Et3N. The reaction mixture was stirred overnight, and washed with 5% aq. citric acid, sat. aq. NaHCO_3_ and brine, and the solvent was evaporated giving a crude solid that was purified on column (eluent: CHCl_3_) giving compound **13a** as a yellow solid, 61% yield after FC; ^1^H NMR (500 MHz, CDCl_3_), δ 8.28 (2d, J = 8.6, 4H, o-NO_2_PhH), 7.90 (s, 2H, 2 × CH = C), 7.60 (2d, J = 8.6 4H, m-NO_2_PhH), 7.25 (m, 5H, PhH), 6.03 (bt, J = 6.1 Hz, 1H, NHCO), 5.29 (t, J = 5.1 Hz, 1H, NHCOO), 5.11 (quint, J = 6.1 Hz, 1H, CHOCO), 4.95 (brs, 1H, NHBoc), 4.24 (m, 1H, CH(NH)CO), 3.17 (m, 4H, 2 × ring CH_2_), 3.12 (m, 2H, CH_2_NHCO), 3.02 (m, 2H, PhCH_2_), 2.94 (m, 2H, CH_2_NHCOO), 1.42 (m, 2H, CH_2_CH_2_CH_2_), 1.40 (s, 9H, Boc) ppm; ^13^C NMR (125 MHz, CDCl_3_), δ 187.46, 172.00, 155.49, 147.50, 141.58, 137.16, 136.53, 134.51, 130.74, 129.20, 128.64, 126.97, 123.77, 80.29, 67.11, 56.05, 38.45, 37.10, 35.78, 33.27, 29.68, 28.22 ppm; ESI-MS, *m*/*z*: 750.3 [M + Na]^+^; 650.1 [M + Na^+^ – CO_2_–C_4_H_8_]. N-Boc deprotection was carried out quantitatively as described above for **7** giving compound **13**. ^1^H NMR (400 MHz, DMSO-d_6_), δ 8.27 (app. d, J = 8.8 Hz, 4H, 2 × o-NO_2_PhH), 8.18 (m, 1H, NHCO), 8.11 (brs, 3H, NH_3_^+^), 7.79 (s, 2H, 2 × CH = C), 7.78 (app. d, J = 8.8 Hz, 4H, 2 x m-NO_2_PhH), 7.26-7.13 (m, 5H, PhH), 7.04 (m, 1H, NHCOO), 5.01 (s, 1H, CHOCO), 3.83 (s, 1H, CH(NH)CO), 3.27-3.13 (m 4H, 2 × ring CH_2_), 2.99-2.84 (m, 4H, PhCH_2_ and CH_2_NHCO), 2.73 (m, 2H, CH_2_NHCOO), 1.29 (m, 2H, CH_2_CH_2_CH_2_) ppm; ^13^C NMR (100 MHz, DMSO-d_6_), δ 187.61, 168.06, 155.62, 147.45, 141.96, 136.23, 135.83, 135.36, 131.68, 129.80, 128.84, 127.46, 124.05, 66.97, 54.01, 38.26, 37.49, 36.77, 33.00, 29.25 ppm. ESI-MS: *m*/*z* 628.1 [M + H]^+^.
*[(3E, 5E)-3,5-bis[(4-nitrophenyl)methylidene]-4-oxocyclohexyl N-[3-[(2R)-2-amino-3-phenylpropanamido]propyl]carbamate (trifluoroacetate salt) (***14***)*. From **7** and N-Boc-(D)Phe, as described above for **13**. Yield: 65%.
*[(3E, 5E)-3,5-bis[(4-nitrophenyl)methylidene]-4-oxocyclohexyl N-[3-[(2S)-2-amino-4-methylpentanamido]propyl]carbamate (trifluoroacetate salt) (***15***)*. N-Boc protected **15a** was obtained from **7** and N-Boc-(L)Leu, as described above for **13**. Yellow solid, 70% yield after FC; ^1^H NMR (500 MHz, DMSO-d_6_), δ 8.28 (app. d, J = 8.7 Hz, 4H, 2 × o-NO_2_PhH), 7.90 (s, 2H, 2 × CH = C), 7.60 (app d, J = 8.7 Hz, 4H, m-NO_2_PhH), 6.38 (bt, J = 6.1 Hz, 1H, NHCO), 5.46 (t, J = 5.0 Hz, 1H, NHCOO), 5.11 (quint, J = 6.0 Hz, 1H, CHOCO), 4.79 (brs, 1H, NHBoc), 4.01 (m, 1H, CHNHBoc), 3.23 (m, 2H, CH_2_NHCOO), 3.18 (m, 4H, 2 × ring CH_2_), 3.09 (m, 2H, CH_2_NHCO), 1.63 (overlapped, 1H, CH(CH_3_)_2_, 1.54 (m, 2H, CH_2_CH_2_CH_2_), 1.43 (overlapped m, 2H), 1.43 (s, 9H, Boc), 0.93 (2d overlapped, J = 6.0 Hz, 6H, CH(CH_3_)_2_) ppm; ^13^C NMR (125 MHz, DMSO-d_6_) δ 187.46, 173.53, 155.50, 147.53, 141.60, 137.19, 134.52, 130.73, 123.78, 80.13, 67.12, 53.35, 40.98, 37.01, 35.68, 33.32, 29.95, 28.25, 24.79, 22.89, 21.93 ppm; ESI-MS, *m*/*z*: 716.3 [M + Na]^+^. N-Boc deprotection of **15a** gave the amine **15** as trifluoroacetate salt; ^1^H NMR (500 MHz), δ 8.32 (t, J = 5.5 Hz, 1H, NHCO), 8.28 (app d, J = 8.7 Hz, 4H, 2 × o-NO_2_PhH), 8.03 (brs, 3H, NH_3_^+^), 7.80 (s, 2H, 2 × CH = C), 7.79 (app d, J = 8.7 Hz, 4H, 2 × m-NO_2_PhH), 7.09 (t, J = 7.0 Hz, 1H, NHCOO), 5.02 (brm, 1H, CHOCO), 3.61 (br, 1H, CH(NH)CO), 3.27–3.15 (m, 4H, 2 × ring CH_2_), 3.02, 2.96 (2m, 2H, CH_2_NHCO), 2.84 (m, 2H, CH_2_NHCOO), 1.57–1.39 (m, 5H, CH(CH_3_)_2_ + CH_2_CH(CH_3_)_2_ + CH_2_CH_2_CH_2_), 0.83 (2d, J = 8.0 Hz, 6H, CH(CH_3_)_2_ ppm; ^13^C NMR (125 MHz, DMSO-d_6_), δ 187.67, 169.13, 155.65, 147.49, 141.98, 136.23, 135.88, 131.68, 124.08, 67.01, 51.43, 40.00, 38.34, 36.88, 32.97, 29.42, 24.07, 22.85, 22.40 ppm. ESI-MS, *m*/*z*: 594.2 [M + H]^+^;

Coupling of 2c with carboxylic acids (Supplementary Materials Figure S5). General Procedure for the synthesis of compounds **16** and **17**. 

To a solution of N-Boc-aminoacid (1.2 mmol) in the minimum amount of DCM, HOBT (0.32 g, 1.2 mmol), EDC (0.46 g, 1.2 mmol), and TEA (0.36 mL, 1.2 mmol) were added in the order, and the mixture was stirred for 30 min. 2c (**1**) (0.76 g, 1.0 mmol) was added in small portions, and the mixture was stirred at 25 °C overnight. The organic phase was washed with 5% aq citric acid, sat. aq NaHCO_3_ and brine, and dried over Na_2_SO_4_. Evaporation of the solvent gave a residue that was purified as indicated.

*[(3E, 5E)-3,5-bis[(4-nitrophenyl)methylidene]-4-oxocyclohexyl (2S)-4-methylpentanoate (***16***).* From **1** and N-Boc-(L)Leu, protected **16a** was obtained after FC, yellow solid, 74% yield; ^1^H NMR (500 MHz, CDCl_3_), δ 8.29 (app d, J = 8.5 Hz, 4H, 2 × o-NO_2_Ph*H*), 7.94 (s, 2H, 2 × C*H* = C), 7.59, 7.57 (2 app. d, J = 8.5 Hz each, 4H, 2 × m- NO_2_Ph*H*), 5.28 (m, 1H, H-4), 4.84 (d, J = 8.0 Hz, 1H, N*H*Boc), 4.14 (m, 1H, C*H*(NH)CO), 3.27–3.08 (m, 4H, CH_2_ ring)), 1.55–1.50 (m, 2H, (CH_3_)_2_CHC*H*_2_)), 1.37 (s, 9H, Boc), 1.36 (overlapped, 1H, (CH_3_)_2_C*H*)), 0.85 (d, J = 6.5 Hz, 6H, (C*H*_3_)_2_CH) ppm; ^13^C NMR (125 MHz, CDCl_3_) δ 186.96, 172.79, 155.28, 147.62, 141.40, 137.67, 133.83, 130.71, 123.83, 80.03, 67.68, 52.09, 41.34, 32.73, 28.19, 24.81, 22.69, 21.84 ppm; ESI-MS, *m*/*z* 594.1 [M + H]^+^. N-Boc deprotection as described for **7** gave the amine **16** as a yellow solid that was purified by precipitation with hexane from an ethyl acetate solution; ^1^H NMR (400 MHz, DMSO-d_6_), δ 8.30-8.27 (broad, 3H, NH_3_^+^), 8.29, 8.28 (2 app d, J = 8.5 Hz, 4H, o-NO_2_Ph*H*), 7.86, 7.84 (2brs, 2H, 2 × C*H*=C), 7.81, 7.77 (2 app d, J = 8.5 Hz, 4H, 2 × m-NO_2_Ph*H*), 5.38 (brm, 1H, C*H*OCO), 3.79 (brt, J = 6.3 Hz, 1H, C*H*NH_3_^+^), [3.39 (dt, J = 3.1 and 17.4, 1H), 3.33 (dt, J = 2.9 and 17.7, 1H), 3.20 (dt, J = 3.7 and 17.7, 1H), 3.19 (dd, J = 4.2 and 17.4 Hz, 1H), 2 × ring CH_2_), 1.44–1.29 (m, 3H, C*H*C*H*_2_), 0.70, 0.69 (2d, J = 6.1 Hz, 6H, (C*H*_3_)_2_CH)) ppm; ^13^C NMR (100 MHz, DMSO-d_6_), δ 187.05, 169.79, 147.54, 141.83, 135.01, 131.77, 124.05, 69.52, 50.77, 32.49, 32.07, 24.32, 22.71, 21.96 ppm; ESI-MS, *m*/*z* 494.2 [M + H]^+^. HRMS, *m*/*z*, found: 494.1923; calcd for [C_26_H_28_N_3_O_7_]^+^: 494.1922.
*[(3E, 5E)-3,5-bis[(4-nitrophenyl)methylidene]-4-oxocyclohexyl (2S)-2-amino-3-phenylpropanoate (***17***).* From **1** and N-Boc-(L)Phe, protected **17a** was obtained after FC as a yellow solid, 68% yield; ^1^H NMR (400 MHz, CDCl_3_), δ: 8.31, 8.29 (2 app. d, J = 8.7 Hz, 4H, 2 × o-NO_2_Ph*H*), 7.91 (2s, 2H, 2 × C*H* = C), 7.57 (2 app. d, J = 8.7 Hz, 4H, 2 × m-NO_2_Ph*H*), 7.11 (m, 3H, Ph*H*), 7.01 (m, 2H, Ph*H*), 5.19 (brm, 1H, C*H*OCO), 4.84 (d, J = 8.2 Hz, 1H, N*H*Boc), 4.45 (q, J = 6.1 Hz, 1H, C*H*(NH)CO), 3.19–2.90 (m, 6H, C*H*_2_Ph and 2 × ring CH_2_), 1.37 (s, 9H, Boc) ppm; ^13^C NMR (100 MHz, CDCl_3_), δ 186.91, 171.36, 154.99, 147.50, 141.56, 137.49, 135.37, 133.73, 130.73, 129.06, 128.37, 127.12, 123.82, 80.27, 67.89, 54.37, 38.07, 32.62, 27.99 ppm. ESI-MS, *m*/*z* 628.3 [M + H]^+^; N-Boc deprotection gave **17** as a yellow solid that was purified by precipitation with hexane from an ethyl acetate solution; ^1^H NMR (400 MHz, DMSO-d_6_), δ, ppm: 8.34–8.27 (overlapped, 3H, NH_3_^+^), 8.33, 8.29 (2 app d, J = 8.7 Hz, 4H, 2 × o-NO_2_Ph*H*), 7.79 (brs, 2H, 2 × C*H* = C), 7.78, 7.76 (2 app d, J = 8.7 Hz, 4H, 2 × m-NO_2_Ph*H*), 7.09–6.99 (2m, 5H, Ph*H*), 5.21 (brm, 1H, C*H*OCO), 4.18 (brs, 1H, C*H*NH_3_^+^), 3.31 (dt, J = 3.5 and 17.0, 1H), 3.20 (dt, J = 4.0 and 16.3, 1H), 3.12 (dd, J = 6.1 and 16.6 Hz, 1H), 2.94 (dd, J = 6.2 and 14.4 Hz, 1H), 2.89 (dd, J = 5.8 and 16.6 Hz, 1H), 2.82 (dd, J = 8.2 and 14.5 Hz, 1H); ^13^C NMR (100 MHz, DMSO-d_6_), δ, ppm: 186.91, 168.97, 147.60, 141.83, 136.58, 134.81, 131.81, 129.48, 128.86, 127.57, 124.08, 69.55, 53.33, 36.53, 32.3; ESI-MS, *m*/*z* 529.2 [M + H]^+^.

### 2.4. Cell Viability

Due to the hydrophobic nature of 2c and its derivatives, the compounds tested were resuspended in DMSO. Cell viability for OVCAR3/Kuramochi was determined by MTT assay in 96-well plates as described, seeding 5 × 10^3^ cells [16,17,18]. Forty-eight hours after seeding, the compounds 2c/derivatives and the inactive control VV1 were administered at different doses. The percentage of viable cells was evaluated at different times after drug administration. 

Patient-derived OC cells (7 × 10^3^ cells/well) were seeded onto a 96-well plate and rested overnight. The following day, the cells were treated in a humidified atmosphere with 2c (2 µM) or VV1 (2 µM) for 72 h at 37 °C in a 5% *v*/*v* CO_2_ incubator. To measure cell viability, cells were incubated with WST-1 regent (Quick Cell Proliferation Kit, Abcam, Cambridge, MA, USA) for 3 h at 37 °C in a 5% *v*/*v* CO_2_ incubator, in a humidified atmosphere. Absorbance was read at 450 nm using the PowerWaveX Select Scanning Microplate Spectrophotometer (Bio-Tek Instruments, Winooski, VT, USA). 

### 2.5. Cell Number 

Cell number for OVCAR3/Kuramochi was determined following cell seeding (7.2 × 10^4^ cells/well in a 6-well plate). Forty-eight hours after seeding, the compounds 2c and the inactive control VV1 were administered at different doses for different time points (12, 18, and 24 h). The number of viable cells was determined using a standard Trypan-Blue exclusion assay, followed by microscopy (Nikon Eclipse TS100, Nikon, Konan, Minato-ku, Tokyo, Japan) visualization using the Thoma counting chamber (Exacta-Optech, San Prospero, Modena, Italy), as we reported [19]. 

### 2.6. Apoptosis

The apoptosis assay was performed by the RealTime-Glo™ Annexin V Apoptosis Assay (Promega, Madison, WI, USA). This is a bioluminescent-based live-cell assay that measures the phosphatidylserine flipped to the outer membrane layer during early apoptosis by Annexin V fusion proteins containing complementary subunits of NanoBiT^®^ luciferase (Annexin V-LgBiT and Annexin V-SmBiT, Promega, Madison, WI, USA). When the two subunits come together due to high levels of phosphatidylserine, they form a functional luciferase able to convert the substrate into the luminescent signal. The luminescent intensity is related to the number of apoptotic cells. OVCAR3 and Kuramochi cells were seeded in 96-well plates at a density of 5.0 × 10^3^ cells/well. After 24 h, cells were treated with increased concentrations of 2c (2–4–10 µM) or VV1 (10 µM) as a negative control coupled to not-treated cells (NT). The detection of the luminescent signal was performed 24 h later, according to the manufacturer’s instructions.

### 2.7. Necrosis

The cytotoxicity assay was performed by the LDH-Glo™ Cytotoxicity Assay (Promega, Madison, WI, USA). This is a bioluminescent-based assay for quantifying lactate dehydrogenase (LDH) released into culture media upon plasma membrane damage. Briefly, the LDH released in culture media catalyzes the oxidation of lactate to pyruvate and the reduction of NAD+ to NADH. The latter is used by reductase to catalyze the reduction of reductase substrate in luciferin, which in turn is converted to a bioluminescent signal by Ultra-Glo™ rLuciferase (Promega). The intensity of luminescence generated is proportional to the amount of LDH in the culture media. OVCAR3 and Kuramochi cells were seeded in 96-well plates at a density of 5.0 × 10^3^ cells/well. After 24 h, cells were treated with increased concentrations of 2c (2–4–10 µM) or VV1 (10 µM) as a negative control coupled to not-treated cells (NT). The quantification of LDH was performed 24 and 48 h after treatment, following the manufacturer’s instructions.

### 2.8. Autophagy

The evaluation of autophagy activity was performed by the Autophagy LC3 HiBiT Reporter Assay System (Promega, Madison, WI, USA). This is a bioluminescent-based assay for autophagy flux detection based on an engineered human LC3 protein fused with a HiBiT tag and a HaloTag^®^ spacer (Promega). The HiBiT is a subunit of the fusion luminescent NanoBiT^®^ enzyme (Promega) complementary to the subunit LgBiT. When the subunits come together, they reconstitute the luminogenic enzyme to convert the specific substrate into the luminescent signal. The working principle of the assay is simple: Initially, the cells are transfected to express the engineered LC3 with the HiBiT tag and then treated with the molecule of interest. At the desired time, the cells are lysed, and the subunits LgBiT with the specific substrate is added to record the luminescent signal. The latter is proportional to the amount of HiBiT subunit in the cell lysate. Since autophagy induction leads to the incorporation and degradation of engineered LC3 with HiBiT, an increase in recorded luminescence intensity is associated with autophagy inhibition. In contrast, an intensity reduction is related to autophagy stimulation. OVCAR3 and Kuramochi cells were seeded on 96-well plates at a density of 5.0 × 10^3^ cells/well. After 24 h, the transfection mixture composed of Lipofectamine 2000 (1 mg/mL, Invitrogen) and 2 µg of Autophagy LC3 HiBiT Reporter Vector (1 mg/mL, Promega) in a weight ratio plasmid/transfectant of 1:2 was administered to cells for 4 h. Thereafter, the transfection medium was replaced with normal growth medium for 3 days. Cells were then treated for 24 h with increasing concentrations of 2c (2–4–10 µM) or VV1 (10 µM) as a negative control coupled to not-treated cells (NT) but transfected. As an additional control, not-transfected and not-treated cells were added (not shown). The detection of the luminescent signal from engineered LC3 was performed 24 h after treatment, following the manufacturer’s instructions.

### 2.9. Cell Cycle Analysis

Cell cycle phase distribution in OVCAR3 was performed as described using the double staining procedure (Ig anti-BrDU and DNA propidium staining) [19]. Briefly, 7.2 × 10^4^ cells/well were seeded in 6-well plates and subjected to treatment with compound 2c or VV1. BrdU (final concentration 10 μM; Becton Dickinson) was administered for 14 h. Then cells were collected, counted, and resuspended in 100 μL of cold 70% EtOH. Subsequently, cells were washed with 1X PBS + 0.5% BSA, centrifuged for 5 min at 3000 rpm, and added to 200 μL of 1M HCl, 0.5% PBS, and 0.5% BSA for 45 min. Afterward, cells were washed in 1X PBS + 0.5% BSA, incubated for 2 min with 0.1M Na borate, followed by a further washing; thereafter, an incubation for 60 min in the dark with an anti-BrdU antibody (BD PharMigen™, San Diego, CA, USA) conjugated to fluorescein or an isotype antibody was performed. After a 1X PBS + 0.5% BSA wash, 100 μL of RNAase, (100 μg/mL of 1X PBS) and PI were added for 1 h. Following two additional washes, cells were analyzed by flow cytometry (Cytomics FC500, Beckman Coulter Inc., Brea, CA, USA).

### 2.10. IC_50_

The determination of the concentration corresponding to 50% cell survival (*IC*_50_) was performed by a two-step procedure. In the first phase, the experimental data concerning the dependence (reduction) of the cell number on the active compound concentration was fitted by means of a straight line, according to the robust fitting strategy as detailed:

*y* = *mx* +*q* (1)

where *y* indicates the number of surviving cells (proportional to the absorbance), *x* is the concentration of the active compound (μM), while *m* and *q* indicate, respectively, the slope and the intercept of the line. The statistical reliability of the fitting was evaluated using the Pearson correlation coefficient *r*.

Since parameter *q* indicates, according to the linear model (Equation (1)), the number of cells that survive when the active compound concentration is zero, the second step of the *IC*_50_ determination involves the normalization of the experimental data with respect to the value of *q*. For greater clarity, the normalized data are multiplied by 100 in order to obtain the percentage of cells that survive an administration of an active compound of concentration equal to *x*. The normalized data were again fitted by means of a straight line according to the robust fitting strategy:

*Y* = *MX* + *Q* (2)

Also in this case, the statistical reliability of the fitting was evaluated using the Pearson correlation coefficient *r*. Knowing *M* and *Q*, the value of the concentration corresponding to the survival of 50% of the cells (*IC*_50_) was determined using the following equation:

(3) $${\mathrm{IC}}_{50}=\frac{50\;-\;Q}{M}$$

Knowing the uncertainties associated with the values of *M* (σ_M_) and *Q* (σ_Q_), deriving from the fitting of Equation (2) on the normalized experimental data multiplied by 100, it was possible to determine the *IC*_50_ uncertainty (σ_CI50_) based on the error propagation law:

(4) $$\sigma_{\mathrm{CI}50}=\sqrt[{2}]{{\left(\frac{50\;-\;Q}{M}\right)}^{2}\sigma_{M}^{2}+{\left(\frac{-1}{M}\right)}^{2}\sigma_{Q}^{2}}$$

### 2.11. Proteasome Activity

The assessment of proteasome activity was performed by Cell-Based Proteasome-Glo™ Assays (Promega, Madison, Wisconsin, USA). This is a bioluminescent-based assay for the independent analysis of the individual protease activity of proteasome: Chemotrypsin-like (CH-L), trypsin-like (T-L), and caspase-like (C-L). This assay is based on three different luminogenic proteasome substrates specific for each type of proteasome individual activity (Suc-LLVY-aminoluciferin for CH-L, Z-LRR-aminoluciferin for T-L, and Z-nLPnLD-aminoluciferin for C-L activity, Promega). The proteasome degradation of substrates leads to the release of aminoluciferin, which can be converted into the luminescent signal by Ultra-Glo™ rLuciferase (Promega). The intensity of luminescent signal generated is related to the activities of each individual protease. OVCAR3 and Kuramochi cells were seeded on 96-well plates at a density of 5.0 × 10^3^ cells/well. After 24 h, cells were treated with increased concentrations of 2c (2–4–10 µM) or VV1 (10 µM) as a negative control coupled to not-treated cells (NT) and with bortezomib (BZB, 0.1 µM) as a positive control. The assessment of proteasome activity was performed 24 h after treatment, following the manufacturer’s instructions.

### 2.12. siRNA Transfection

For uptake studies, OVCAR3/Kuramochi were seeded at a density of 7.2 × 10^4^ cells/well in 6-well plates on a collagen I-coated slide; transfection was carried out for 4 h using the red fluorescent Trilencer siRNA (6 µL, OriGene Technologies GmbH, Herford, Germany) complexed with lipofectamine 2000 (2.5 μL, ThermoFisher Scientific). Following transfection, cells were fixed in 4% paraformaldehyde (Sigma-Aldrich) and stained with DAPI; images were then taken by a Leica DM2000 fluorescence microscope (20 × objective). The sequences of the control siRNA (siGL2) and the anti-E2F1 siRNA (siE2F1), as well as the transfection procedure, were substantially as described [20]. To evaluate OC cell viability following siRNA transfection, the day before transfection, OC cells were seeded at a density of 5 × 10^3^ cells/well in a 96-well plate in 100 μL of complete medium. Optimal transfection conditions were obtained by using Lipofectamine 2000 (1 mg/mL, ThermoFisher Scientific) at a weight ratio of siRNA–transfectant of 1:2. The mixture of liposome–siRNA (220 nM) was then administered to the cells for 4 h at 37 °C in the presence of serum-free medium Optimem (ThermoFisher Scientific). Afterward, the transfection medium was removed, cells were washed in PBS, and then 100 μL of complete medium were added to the cells. Viability, measured by the MTT test, was evaluated 2 and 3 days thereafter.

### 2.13. Western Blot Analysis 

Protein extraction was performed as described [18]. Briefly, 40 μg of protein extract was resolved onto 12% (E2F1) or 8% (PARP) SDS-PAGE and blotted onto a 0.22 µm nitrocellulose membrane (Schleicher & Schuell, Keene, NH, USA). The rabbit polyclonal antibody anti-E2F1 (Santa Cruz, dilution 1:100) and the mouse monoclonal antibody anti-PARP-1 (BD Pharmigen, dilution 1:1000) were used. The loading control protein GAPDH (Santa Cruz, dilution 1:1000) was probed on the same membrane. Blots were developed using the corresponding secondary horseradish peroxidase antibodies (Santa Cruz) by the enhanced chemiluminescence detection system (Pierce) and exposed to Kodak film (Sigma-Aldrich).

### 2.14. RT-PCR

Extraction of total RNA from cell pellets and quantification of RNA were performed as previously described [18]. The primers (Eurofins Genomics) and real-time amplification conditions for E2F1 and 28S RNA have been highlighted previously [18]. For the amplification of UCHL5 ubiquitin C-terminal hydrolase L5 (UCHL5), the following primers were used: forward 5′GAGTGGTGCCTCATGGAA3′ and reverse 5′AACCCATGAACTGGCTTTAAT3′; amplification conditions were performed according to the PowerUp^TM^ Syber GREEN master MIX (Fast cycling Mode for TM ≥ 60 °C): denaturation 95 °C for 3 s, annealing 60 °C for 30 s for 40 cycles. As thermocycler, we used the Applied Biosystem StepOnePlusTM real-time PCR.

### 2.15. Patient Derived Organoids (PDO)

Organoids were derived from completely anonymized specimens, and prior informed consent for research purposes was obtained from the biobank to collect the samples at the National Cancer Institute (CRO) of Aviano. Ascitic fluids were subjected to centrifugation at 1000 rpm for 10 min, and the resulting cell pellets were washed twice with HBSS (Gibco, Waltham, MA, USA). To remove erythrocytes present in the fluid, cold red blood cell lysis buffer (Roche Diagnostics, Basel, Switzerland) was added and kept on ice under gentle stirring for 10 min. The pellet was then centrifuged again at 1000 rpm for 10 min and resuspended in Cultrex RGF BME, Type 2 (Bio-techne, Minnesota, MN, USA). The solid tumor tissue was incubated in Dulbecco’s modified Eagle’s medium/Nutrient Mixture F-12 Ham supplemented with Levofloxacin 100 μg/mL, Vancotex 25 μg/mL, Ciproxin 5 μg/mL, Gentamicin 200 μg/mL, and Fungizone 5 μg/mL for 30 min. The tissues were finely minced into approximately 0.5 mm diameter pieces using dissection scissors, and then 1 mL of 4 mg/mL collagenase IV solution (Gibco, Waltham, MA, USA) was added. The mixture was incubated at 37 °C for 45 min and mechanically dissociated by pipetting. The resulting clusters of cells were subjected to centrifugation at 1000 rpm for 10 min, resuspended in an appropriate volume of Cultrex RGF BME, Type 2 (Bio-techne, Minneapolis, MN, USA), and seeded onto a 24-well cell culture plate. Following the polymerization of Cultrex, 500 μL of organoid culture medium, as described, was added to each well and refreshed every 3 days [21].

The patient-derived organoids (PDOs) were cultured according to the supplier’s instructions at 37 °C and 5% CO_2_. Clusters of PDOs were mixed in Cultrex RGF BME, Type 2 (Bio-techne, Minneapolis, MN, USA), and 2 μL of this mixture were seeded in 96-well plates. The seeded PDOs were then treated with four different concentrations of VV1 and 2c (10, 4, and 2 μM) in three replicates, along with a non-treated control condition. Due to the hydrophobic nature of 2c and its derivatives, the compounds tested were resuspended in DMSO. Cell viability was assessed at 12, 16, and 24 h using CellTiter-Glo 3D (Promega, Madison, WI, USA) with BioTek Synergy H1.

### 2.16. In Vivo Tests

OVCAR3 cells (1 × 10^7^cells/100 μL of physiological saline containing 50% Matrigel, Corning) were used to generate the subcutaneous xenograft mouse model of OC in SCID mice (Envigo, Indianapolis, Indiana, Italy). All in vivo experiments were performed according to the guidelines of the EU Directive (2010/63/EU) and the approval of the Veterinary Administration of the Ministry of Agriculture, Forestry and Food of the Republic of Slovenia (approval no. U34401-23/2018/5). Due to the hydrophobic nature of 2c and its derivatives, the compounds tested were resuspended in DMSO. When the longest diameter of the tumors reached 6 mm (approximately 40 mm^3^), the mice were randomly divided into different groups and subjected to drug administration by intra-tumor injection (4 animals treated with one dose of 1 μg 2c, 4 animals treated with one dose of 1 μg VV1). The mice were humanely killed by CO_2_ when the tumor volume reached 70 mm^3^. Considering the initial tumor volume of approximately 40 mm^3^, the dose of 1 μg 2c corresponds to an estimated initial intra-tumor concentration of 40 μM. The growth of the tumors was evaluated by a simple but powerful mathematical model we previously developed [19]. 

### 2.17. Mathematical Modelling of 2c Effects on Tumor Growth In Vivo

In order to accurately describe the effects of 2c on tumor growth, a recent mathematical model aimed at describing the increase in tumor volume in vivo was used [18]. This model is based on the assumption that tumor volume, and thus the number of tumor cells (*N*), increases according to an exponential evolution. To account for the presence of external confounding factors (e.g., anti-tumor drugs), it is assumed that the proportion (*f*) of cells that can divide depends on time (*t*) according to the following exponential law:

(5) $$f=\left(f_{0}{\;-\;f}_{\infty }\right)e^{-k_{f}t}{+f}_{\infty }$$

where *f*_0_ and *f*_∞_ are, respectively, the initial (*t* = 0) and the final (*t* → ∞) fraction of cells that undergo division and *k*_f_ is a kinetic constant connected to the kinetics of *f* decrease. Thus, *f* can be viewed as a correcting factor for the classical kinetics equation describing the time increase in the tumor cells number *N*:

(6) $$\frac{dN}{dt}{=k}_{D}N\left(\left(f_{0}{\;-\;f}_{\infty }\right)e^{-k_{f}t}{+f}_{\infty }\right)=V_{D}$$

where *k*_D_ is the kinetic constant connected to cells division speed *V*_D_ (cells/time). Equation (6) analytical solution is:

(7) $$\frac{N}{N_{0\;}}{=\exp(k}_{D}{((f}_{0}{\;-\;f}_{\infty })(\frac{{1\;-\;e}^{-k_{f}t}}{k_{f}}{)+f}_{\infty }t))$$

where *N*_0_ is the initial number of tumor cells. In data analysis we assumed *f*_0_ = 1 (100% of the cells can undergo division) and *f*_∞_ = 0 (after a very long time cells cannot undergo division). The combination of Equations (5) and (7) provides the expression for the velocity of tumor volume increase:

(8) $$\frac{V_{D}}{N_{0\;}}{=V}_{D}^{+}{=k}_{D}{\;*\;\exp(k}_{D}{((f}_{0}{\;-\;f}_{\infty })(\frac{{1\;-\;e}^{-k_{f}t}}{k_{f}}{)+f}_{\infty }t))\;*\;\left(\left(f_{0}{\;-\;f}_{\infty }\right)e^{-k_{f}t}{+f}_{\infty }\right)$$

Equation (8) expresses the number of cells generated by each original cell after time *t*.

### 2.18. Molecular Modelling 

The calculations were carried out using either the Schrodinger or the Yasara molecular modelling suites. Optimizations of the proteins were performed in the AMBER14 force field. Molecular dynamics were carried out in the NTP ensemble at 298 °K, inside a periodic box of explicit TIP4P water molecules, at pH 7.4 and 0.9% NaCl. Dynamics were run for 10 ns (1.25 fs timestep). The geometries and charge distributions of the ligands were obtained at the B3LYP-6-31G** level. Non-covalent docking was carried out with the VINA module implemented in Yasara, while for covalent docking, the starting geometries of the Michael adducts were manually built inside the catalytic site of the enzyme. All the models were built starting from the crystallographic structure of UCHL5. The geometry of the catalytic domain of the enzyme was downloaded from the Protein Data Bank, structures 3RII and 3RIS [22]. The missing amino acids between Gln146 and Ala157 were added, and the starting geometry for this loop was assigned by carrying out a homology comparison with similar sequences in the PDB. The model was then thermalized with a dynamic run and reoptimized for further docking studies.

### 2.19. Statistical Analysis

Statistical analysis was performed using GraphPad Prism 8. All data, expressed as the mean ± SEM, were tested for normal distribution using the Shapiro–Wilk test. *P* values were calculated using the unpaired or paired t test with or without Welch correction, the Mann–Whitney test or the Wilcoxon matched-pairs signed-ranks test, as appropriate. *p* values < 0.05 were considered statistically significant.

## 3. Results

### 3.1. Uptake Studies

The effects of 2c (Figure 1A left) were studied in OVCAR3 cells, which represent a commonly used model of OC. We also considered Kuramochi cells, which have the highest genetic similarity with HGSOC among the available OC cell lines [12]. Fluorescently labelled 2c (2c-F) was used to study cellular uptake. Our data (Figure 1B,C) clearly indicate a time-dependent uptake already readily visible 6 h after treatment that almost extended to the entire cell population 24 h after 2c-F administration. In addition, confocal microscopy analysis proved that 2c-F entered the cells (Figure 1D,E). 

Similar uptake was observed for the inactive version of 2c (VV1, see below) used as a negative control in the tests reported in the following sections (Figure S1); moreover, two additional derivatives of 2c (number 10 and 11, see Table 1), which had no or excellent activity, respectively, resulted in similar uptake (Figure S1) to VV1/2c.

### 3.2. Cell Viability, Cell Number

**Figure 2:**
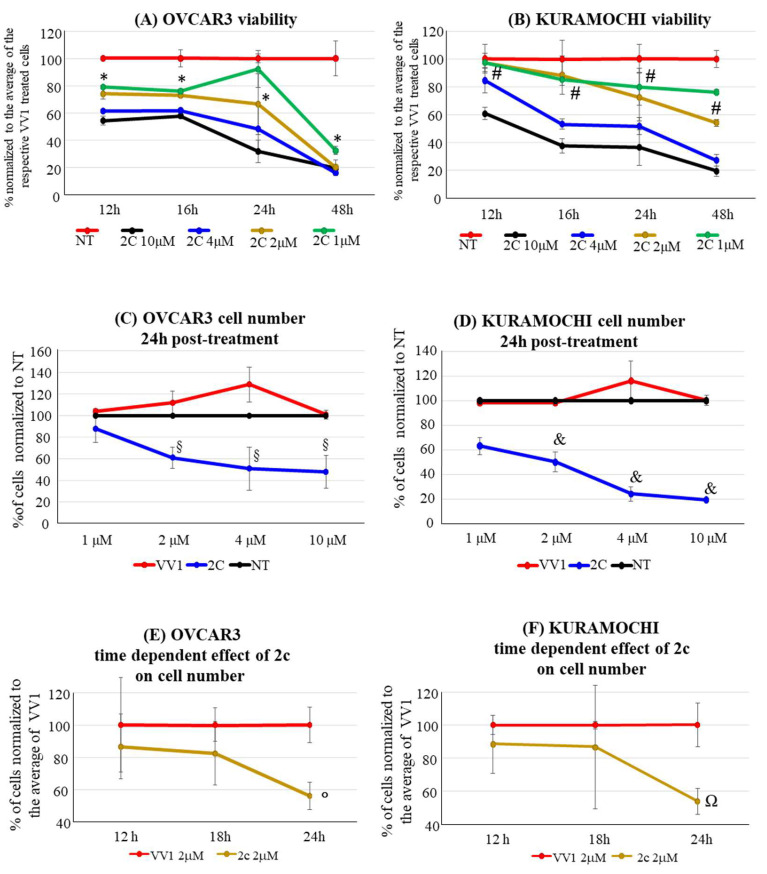
Effect of 2c on cell viability and number. (**A**) The viability of OVCAR3 and (**B**) Kuramochi treated by 2c or VV1 using different drug concentrations and analysis time was evaluated. Data, normalized to the average of the respective VV1, are reported as mean ± SEM, n = 10; (* *p* = 0.0001 for 1-2-4-10 μM 2c 12 h vs. VV1 12 h, 1-2-4-10 μM 2c 16 h vs. VV1 16 h, 2-4-10 μM 2c 24 h vs. VV1 24 h; # *p* = 0.021 for 4-10 μM 2c 12 h vs. VV1 12 h, 1-2-4-10 μM 2c 16 h vs. VV1 16 h, 2-4-10 μM 2c 24 h vs. VV1 24 h). (**C**) Cell number of OVCAR3 and (**D**) Kuramochi following 24 h administration of different 2c concentrations. Data, normalized to the average of NT (non-treated cells), are represented as mean ± SEM, n = 6; (^§^ *p* < 0.028 for 2c 10-4-2 μM vs. VV1 10-4-2 μM; ^&^ *p* < 0.028 for 2c 10-4-2 μM vs. VV1 10-4-2 μM). (**E**) Cell number of OVCAR3 and (**F**) Kuramochi using 2 μM of 2c at different times after administration. Data, normalized to the average of VV1-treated cells, are represented as mean ± SEM, n = 3; (° *p* = 0.0084 for 2c 24 h vs. VV1 24f; ^Ω^ *p* = 0.023 for 2c 24 h vs. VV1 24 h).

In OVCAR3 and Kuramochi, 2c displayed a time- and dose-dependent effect on cell viability (Figure 2A,B). As a control, an inactive version of 2c was used (VV1, Figure 1A right). The presence of the -OH group (Figure 1A right) prevents the interaction with the DUBs cysteine group. Cell counting confirmed the dose- (Figure 2C,D) and time-dependent (Figure 2E,F) effects of 2c. Notably, we observed that the fluorescently labelled 2c used for uptake studies displayed no activity; we believe this depends on the steric hindrance of the fluorochrome, which may impair 2c docking to the catalytic site of the target enzyme.

The microscopic examination of the 2c-treated cells showed (Figure S2) a widespread cytoplasmic vacuolization, possibly as a consequence of ER stress, cell volume alteration, and cell membrane deformation; these morphological alterations resemble those of apoptotic cells [9,23]. Thus, the possible pro-apoptotic effect of 2c was investigated.

### 3.3. Apoptosis, Necrosis, Autophagy and Cell Cycle

**Figure 3:**
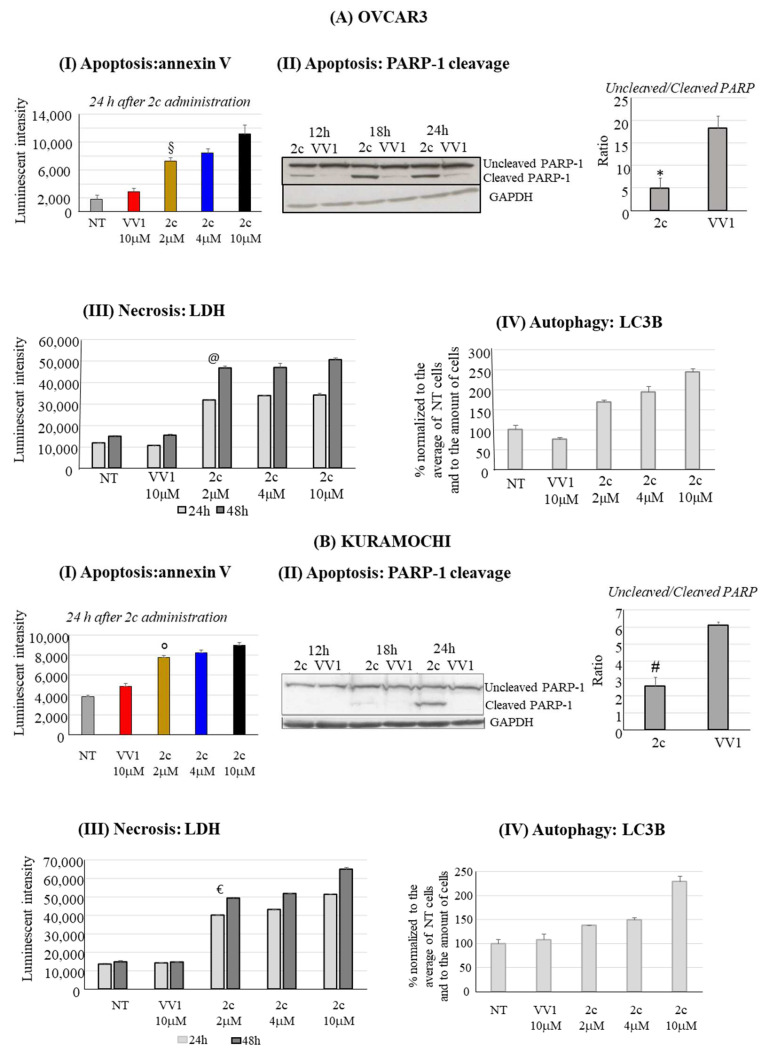
Apoptotic effects of 2c. OVCAR3 and Kuramochi were treated by 2c at different concentrations (**AI** and **BI**, respectively) and analyzed for the externalization of the apoptotic marker annexin V, 24 h after drug treatment; data are reported as mean ± SEM, n = 4, ^§^ *p* = 0.028 2c (all concentrations) vs. VV1-treated cells, ° *p* = 0.028 2c (all concentrations) vs. VV1-treated cells. The cleavage of the apoptotic marker PARP-1 was also analyzed in OVCAR3 (**AII**) and Kuramochi (**BII**) treated with 2c 2 μM at different time points; on the left a representative Western blot is shown; on the right the ratio uncleaved/cleaved PARP-1 is reported for 2c and in VV1 (control) treated cells considering all the time points evaluated; data are reported as mean ± SEM, n = 8, * *p* = 0.0048 2c vs. VV1-treated cells, # *p* = 0.0001 2c vs. VV1-treated cells. NT = non-treated cells. Necrotic effects of 2c. OVCAR3 and Kuramochi were treated by 2c at different concentrations (**AIII** and **BIII**, respectively) and analyzed for the release of the necrotic marker LDH, 24 and 48 h after drug treatment; data are reported as mean ± SEM, n = 4, ^@^ *p* = 0.028 2c (all concentrations) vs. VV1-treated cells, ^€^ p = 0.028 2c (all concentrations) vs. VV1-treated cells. NT = non-treated cells. *Effect of 2c on autophagy*. OVCAR3 and Kuramochi were treated by 2c at different concentrations (**AIV** and **BIV**, respectively) and analyzed by the autophagy LC3 HiBiT Reporter Assay System 24 h after drug treatment; data are reported as mean ± SEM, n = 4. NT = non-treated cells.

The apoptotic effect of 2c was investigated by evaluating the externalization of the apoptotic marker annexin V. Our data show an evident dose-dependent apoptotic effect in OVCAR3 (Figure 3AI) and in Kuramochi cells (Figure 3BI). The pro-apoptotic effect was confirmed by evaluating the cleavage of poly (ADP-ribose) polymerase-1 (PARP-1), another known marker of apoptosis, in both OVCAR3 (Figure 3AII) and Kuramochi (Figure 3BII). 

In parallel to the pro-apoptotic effect of 2c, we also analyzed the possible induction of two other forms of cell death, i.e., necrosis and autophagy. Our data show a significant contribution of necrosis to the cell death induced by 2c in OVCAR3 (Figure 3AIII) and Kuramochi (Figure 3BIII). In both cell lines, the effect is clearly evident already 24 h after the treatment with 2 μM of 2c. Notably, at the highest concentration tested (10 μM), the effect is quantitatively superior in Kuramochi compared to OVCAR3. In contrast, autophagy was inhibited (Figure 3AIV,BIV) in both cell lines as revealed by the Autophagy LC3 HiBiT Reporter Assay System; indeed, an increase in recorded luminescent intensity is associated with autophagy inhibition (see M&M for technical details). 

**Figure 4:**
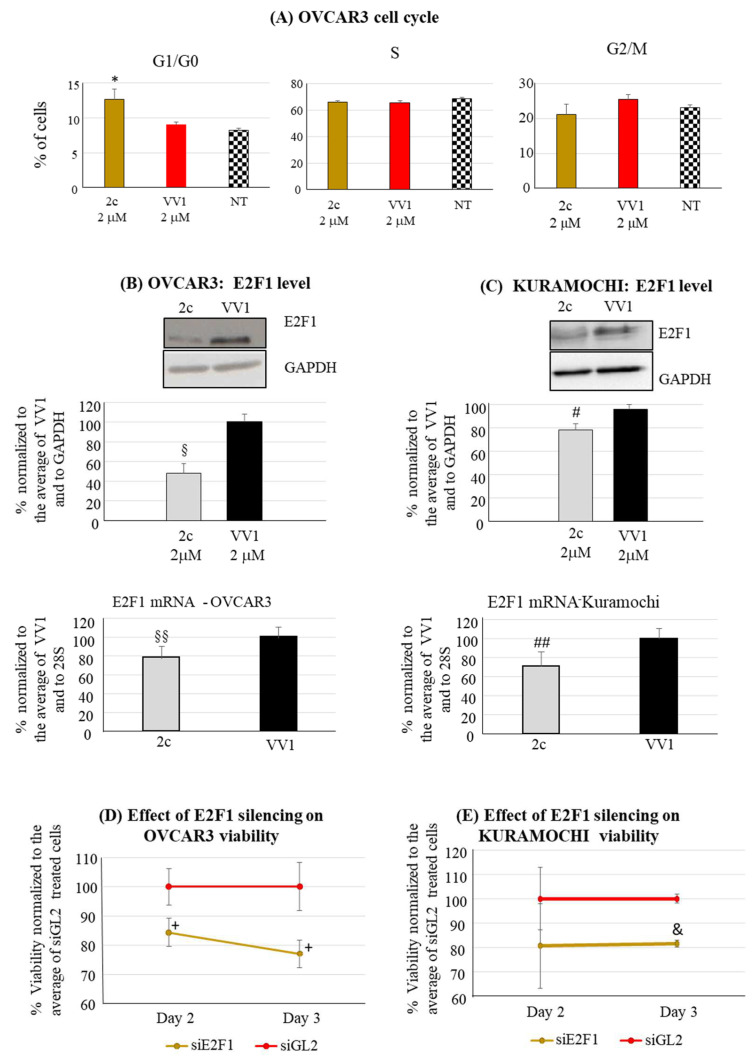
Effect of 2c on cell proliferation. (**A**) The effects of 2c (2 μM) on cell cycle phase distribution in OVCAR3 are evaluated 24 h after drug administration. The data for each cell cycle phase are reported as the % of total cells referred to all cell cycle phases. As control, VV1-treated cells and non-treated cells (NT) are evaluated; data are shown as mean ± SEM, n = 4; * *p* < 0.042 2c-treated cells vs. VV1-treated cells and NT. In (**B**) OVCAR3 and in (**C**) Kuramochi protein levels (up) and mRNA levels (down) of the transcription factor E2F1 24 h after 2c (2 μM) administration are reported; as control VV1-treated cells are included; data are shown as mean ± SEM, n = 5; ^§^ *p* = 0.003 for 2c-treated cells vs. VV1-treated cells, # *p* = 0.03 for 2c-treated cells vs. VV1-treated cells; n = 6; ^§§^ *p* = 0.011 for 2c-treated cells vs. VV1-treated cells; ## *p* = 0.0003 for 2c-treated cells vs. VV1-treated cells. In (**D**) for OVCAR3 and in (**E**) for Kuramochi cell line, the effects of E2F1 silencing by siRNA (siE2F1) are reported. siE2F1 (220 nM) was delivered to 96-well-plate cultured cells; the effects on cell viability were evaluated by MTT test 2 and 3 days after siE2F1 delivery; as control, cells treated by the siRNA siGL2 (siRNA against the luciferase mRNA, 220 nM) were considered; the data, expressed as % of the average of cells treated by siGL2, are shown as mean ± SEM, n = 15; + *p* < 0.047, compared to siGL2-treated cells, ^&^ *p* = 0.0001 compared to siGL2-treated cells.

Finally, we focused on 2c impact on cell cycle phase distribution. For technical reasons, the OVCAR3 cell line was considered the most suited for the test. 2c significantly increased the number of G1/G0 (Figure 4A) compared to the control VV1-treated cells or untreated cells (NT). Cell accumulation in G1/G0 prompted us to investigate the effects on the expression of G1/S transition drivers. Among these, we observed a significant decrease in the protein and mRNA level of E2F1 both in OVCAR3 (Figure 4B) and Kuramochi (Figure 4C); E2F1 is a well-known promoter of the G1/S transition and is also implicated in OC [24]. To determine the functional involvement of E2F1 in the 2c-mediated impairment of the cell cycle, E2F1 was silenced by a specific siRNA (siE2F1). Following an effective uptake of the siRNA in both OVCAR3 and Kuramochi (Figure S3), we could show that the targeting of E2F1 resulted in a decrease in OVCAR3/Kuramochi viability (Figure 4D,E) compared to a control siRNA (siGL2). 

### 3.4. Effect of 2c In Vivo and in Patient-Derived Cells

**Figure 5:**
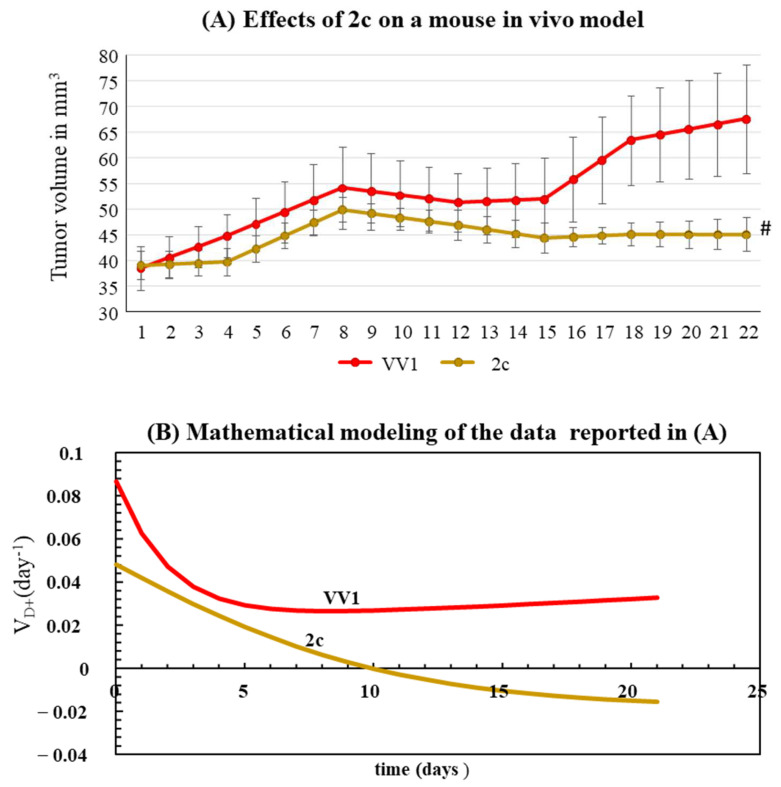
Effect of 2c in an in vivo mouse model of OC. (**A**) Effects of 2c on tumor growth; the drug was administered on day 0; data are reported in mm^3^ as mean ± SEM; as control, VV1-treated animals are reported; ^#^ *p* = 0.0001 for 2c-treated vs. VV1-treated animals. (**B**) Data interpretation through the developed mathematical model reveals that 2c determines a reduction in tumor cell proliferation (VD^+^ = number of cells generated in one day from each original cell) that, after ten days, becomes negative, predicting the stop of tumor cell growth over time (fitting parameters: VV1 => K_D_ (day^−1^) = 0.087, *K_f_* (day^−1^) = 0.547, *f*_∞_^+^ = 0.222; 2c => *K_D_* (day^−1^) = 0.048, *K_f_* (day^−1^) = 0.129, *f*_∞_^+^ = −0.375).

OVCAR3 was used to generate a subcutaneous xenograft mouse model of OC. Upon the tumor reaching a volume of about 40 mm^3^, we injected intratumorally a single dose of 1 μg of either 2c or VV1 (control) into the tumor. This corresponded to an estimated initial intra-tumor concentration of 40 μM, greater than the doses tested in vitro. This concentration was chosen in consideration of the 2c drainage (by blood), which can occur in vivo. Compared to VV1-treated control animals, 2c was able to delay tumor growth significantly (Figure 5A). To gain deeper insight into the effect of 2c on tumor growth, we used a simple but powerful mathematical model we previously developed [19]. The mathematical model, built to describe/predict the growth of tumor cells in animal models, was fitted to the data shown in Figure 5A. This procedure allowed us to determine the increase in tumor volume using VD^+^, which expresses the number of cells generated in one day from each original tumor cell (Figure 5B). The mathematical analysis shows that VD^+^ is always >0 in control animals, indicating a progressive and continuous tumor growth; in animals treated with 2c, VD^+^ assumes negative values from ten days on, thus predicting the progressive tumor-shrinking over time. 

**Figure 6:**
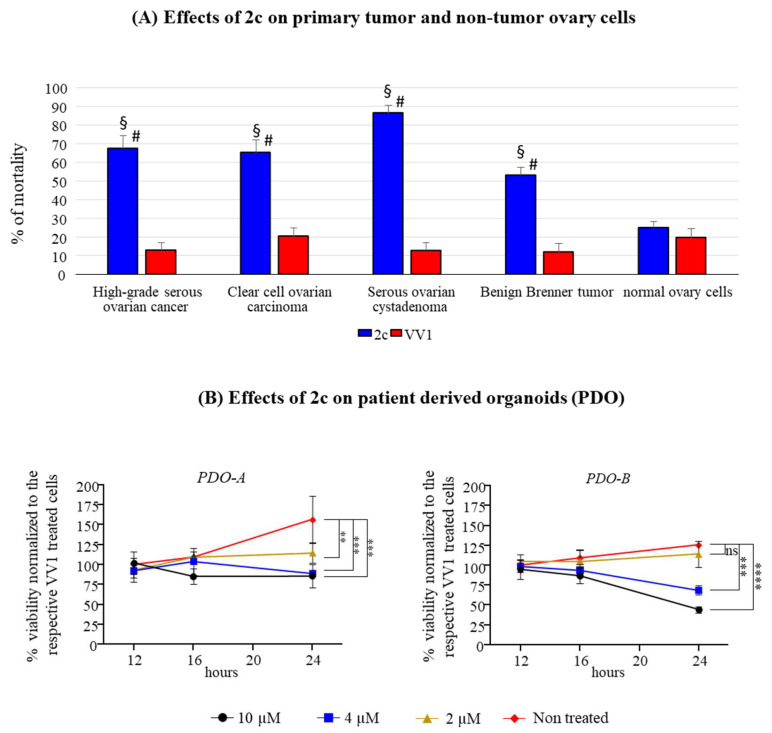
Effect of 2c on patient-derived OC cells. (**A**) Effects of 2c (2 μM) on the viability of ovary tumor/non-tumor cells isolated from patients, evaluated 72 h after drug administration. As a control, VV1-treated cells are included; data are reported as mean ± SEM, n = 12 (HGSOC), n = 9 (clear cell ovarian carcinoma), n = 6 (serous ovarian cystadenoma), n = 6 (benign Brenner tumor), or n = 6 (non-tumor ovary cells), ^§^ *p* < 0.0005 mortality induced by 2c in tumor cells vs. non-tumor cells; ^#^ *p* < 0.0001 2c-treated tumor cells vs. VV1-treated tumor cells. (**B**) Viability of PDO-A and PDO-B-treated by 2c or VV1 using different drug concentrations and time of analysis; data, normalized to the average of the respective VV1, are reported as mean ± SD, n = 3; ** *p* ≤ 0.01, *** *p* ≤ 0.001, and **** *p* ≤ 0.0001 compared to non-treated cells. ns = non significant.

To extend the significance of 2c efficacy, its effect was tested in a panel of patient-derived OC cells cultured in 2D. These included HGSOC (4 patients), clear cell ovary carcinoma (3 patients), serous ovarian cystadenoma (2 patients), and benign Brenner tumor (2 patients). The effects on all OC types are reported in Figure 6A. Our data confirmed the effectiveness of 2c in inducing cell death compared to the control VV1-treated cells. Remarkably, 2c had far fewer effects on non-tumor ovary cells, suggesting a tumor-specific effect.

The 2c effect has also been evaluated on two HGSOC patient-derived organoids (PDO). PDO-A is an ascite-derived from HGSOC, while PDO-B represents an HGSOC chemonaive tumor. All the patients were responsive to platinum-based chemotherapy. In both cases, it is possible to observe a dose-dependent effect on PDOs, with PDO-B being more affected than PDO-A (Figure 6B).

### 3.5. 2c Relation with Proteasome Activity and UCHL5

**Figure 7:**
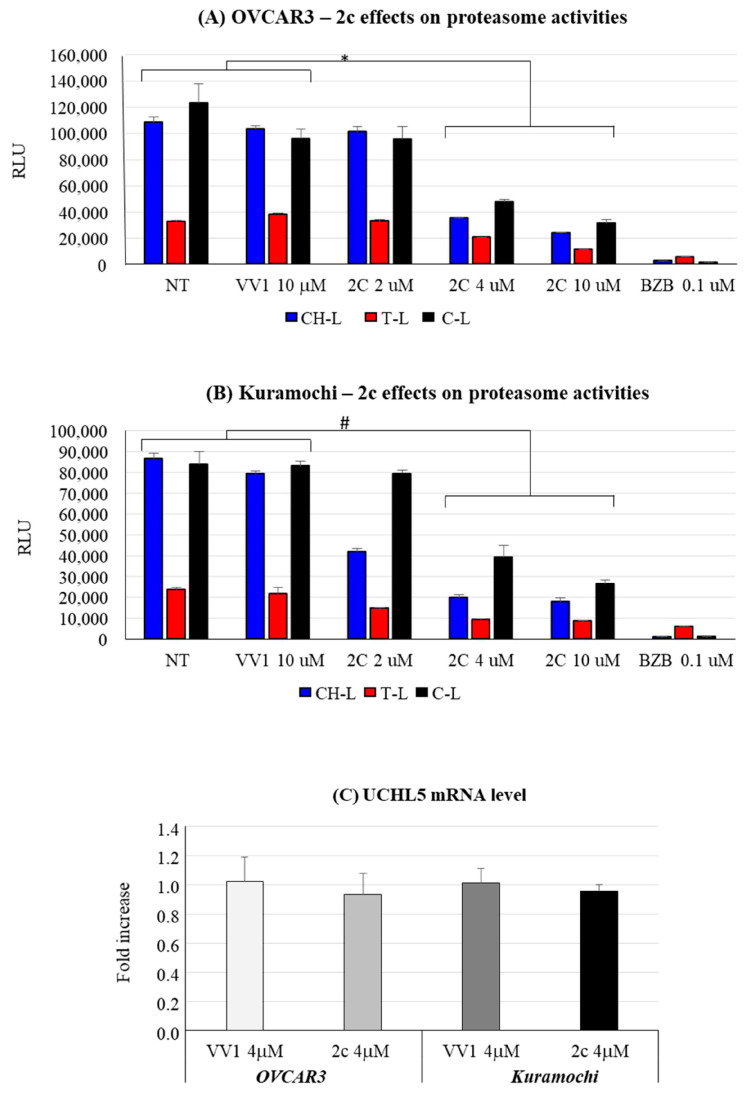
Effect of 2c on proteasome activity and on the mRNA level of deubiquitinase UCHL5. The different proteasome enzymatic activities (chemotrypsin-like (CH-L), trypsin-like (T-L), and caspase-like (C-L)) were evaluated in OVCAR3 (**A**) and Kuramochi (**B**) after the administration of increasing concentrations of 2c, 24 h after drug administration; y axis: relative light unit (RLU), directly proportional to the extent of the enzymatic activity; BZB = bortezomib, used as positive control of proteasome inhibitor; data are reported as mean ± SEM, n = 4; * *p* < 0.0001, and ^#^ *p* ≤ 0.0001 compared to non-treated cells and VV1-treated cells. (**C**) The mRNA level of UCHL5 has been evaluated 24 h after 2c administration; y axis: fold increase calculated on the basis of the ddct method; VV1: inactive control for 2c; data, normalized to 28S rRNA, are reported as mean ± SEM, n = 4.

To substantiate the concept that the effects exerted by 2c on OC cells are related to an effect on the proteasome–ubiquitin system, we explored the 2c effect on proteasome enzymatic activities and on the deubiquitinase UCHL5, which we previously identified as a 2c target [6]. In both cell lines (Figure 7A,B), we observed a dose-dependent reduction in the three proteasome enzymatic activities, i.e., chemotrypsin-like (CH-L), trypsin-like (T-L), and caspase-like (C-L), by 2c; as a positive control, we tested the drug bortezomib, known to be a potent proteasome inhibitor [25]. Importantly, our data show that UCHL5, a target of 2c, is expressed in both cell lines (Figure 7C). Notably, 2c does not affect the expression level of UCHL5, supporting the concept that it may impair UCHL5 activity through a direct interaction (see also below). 

### 3.6. In Silico Docking of 2c to Deubiquitinase UCHL5

**Figure 8:**
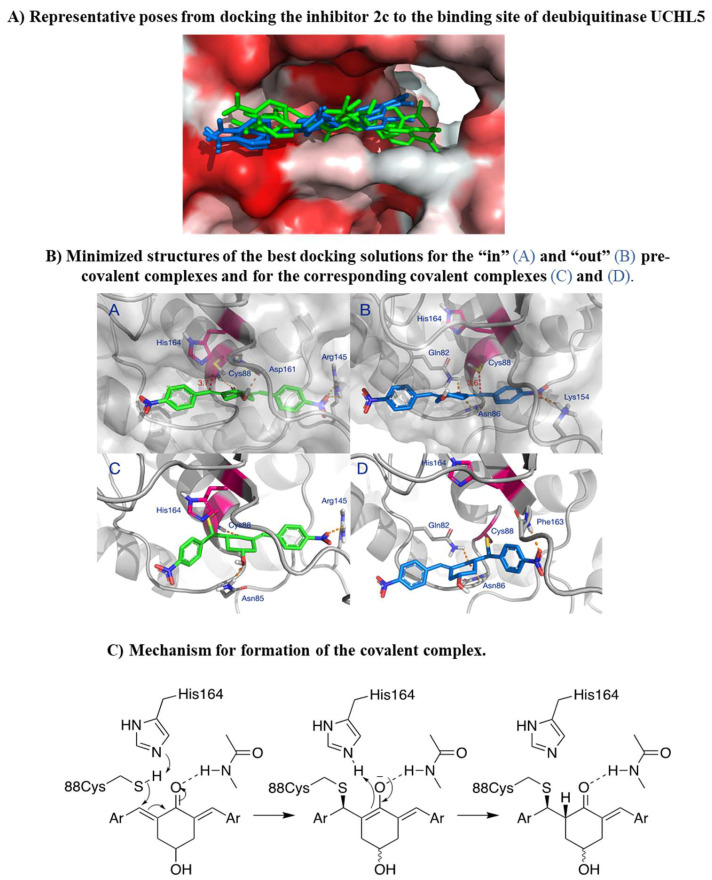
In silico docking of 2c to deubiquitinase UCHL5. (**A**) Representative poses from docking the inhibitor 2c to the binding site of deubiquitinase UCHL5. “In” poses are colored green and “out” poses are colored cyan. The protein’s surface is colored according to hydrophobicity (red: hydrophobic; white: hydrophilic). (**B**) Minimized structures of the best docking solutions for the “in” (A) and “out” (B) pre-covalent complexes and for the corresponding covalent complexes (C) and (D). The inhibitor 2c is shown in green and blue, respectively; the catalytic dyad is shown in magenta. Hydrogen bonds and main polar interactions are indicated by orange dashes. Distances between the sulfur atom of the catalytic cysteine and the electrophilic carbon are in red. (**C**) Mechanism for the formation of the covalent complex.

Following the demonstration of 2c effectiveness in different relevant OC models, we wanted to explore in silico the molecular interaction of 2c with its target. Deubiquitinase UCHL5 was considered as we previously identified this as a possible 2c target and as it is expressed in our cellular models of OC [6]. The catalytic cysteine of UCHL5 lies at the bottom of a narrow hydrophobic crevice, approximately 30 Å long. Docking 2c to this site reveals two possible binding modes for the initial non-covalent complex that precedes the formation of the C-S bond (Figure 8A). In the “in” orientation (green in Figure 8A), 2c is deeply inserted into the crevice, with one aromatic group engaging the tunnel leading to the ubiquitin-binding site while the other aromatic group is more exposed. In the “out” orientation (blue in Figure 8A), the inhibitor is shifted 4 ÷ 5 Å away from the tunnel leading to the ubiquitin binding site. 

In the optimized “in” complex, the inhibitor’s carbonyl is hydrogen bonded to Cys88, and a second hydrogen bond is present between the OH group and the backbone carbonyl of Asp161 (Figure 8B panel A). A favorable electrostatic interaction is established between (Figure S4A) the nitro group and Arg145, and other contacts are made with the side chains of Leu10, Asn85, Ala87, His164, Leu181, and with the hydrophobic portion of Gln82. This brings the outer electrophilic carbon in close proximity to the catalytic thiol group of Cys88, ideally aligned for addition to the double bond (Figure 8B panel A). In the optimized “out” complex, a hydrogen bond is established between Asn86 and the inhibitor’s carbonyl, and the latter is at hydrogen bond distance with the side chain of Gln82 (Figure 8B panel B). The polar interaction between the inner nitro group and Arg145, in the “in” complex, is replaced by a similar interaction with the ε-amino group of Lys154. The inhibitor is also in contact with the side chains of Trp58, Asn85, Ala162, and Phe163 (Figure S4B). This results in comparable binding energies for the two poses. The 4÷5 Å displacement of the inhibitor in the “out” complex brings the thiol group of Cys88 in proximity to the inhibitor’s inner electrophilic carbon, at approximately the same distance as in the “in” complex (Figure 8B panel B). A molecular dynamics simulation shows that, at room temperature, the inhibitor can freely move inside the binding site, exchanging orientation, without the collapse of the complex. As a result, the catalytic SH of Cys88 can interact with either electrophilic carbon of 2c, which behaves effectively as a divalent inhibitor. This might explain why 2c is so efficient as an inhibitor of cysteine enzymes such as UCHL5. 

### 3.7. In Silico Modelling of Covalent Complexes

The covalent complexes resulting from the reaction of the thiol group of Cys88 with the electrophilic carbons of 2c are shown in Figure 8B, panels C and D. The most favorable polar interactions are retained in the covalent complexes. However, the structure of the inhibitor is considerably bent because of the formation of the covalent bond and the rehybridization of the β-carbon. In the “in” complex, this results in the loss of the interaction between the nitro group and Lys154 (Figure 8B, panel C), which is replaced by an interaction of the same nitro group with the backbone NH of Phe163 (Figure 8B, panel D). 

The molecular modelling studies of this work also allow us to propose a model for the irreversible inhibition of deubiquitinase UCHL5, a privileged target of 2c. In the pre-covalent complex (left), deprotonation of the catalytic thiol by the histidine residue of the catalytic dyad initiates the reaction with the inhibitor, as shown in Figure 8C. The hydrogen bonds between the carbonyl and the enzyme lock the inhibitor in a favorable orientation for attack by the nucleophilic cysteine and, presumably, stabilize the intermediate enolate. Addition occurs on the Re face of the outer electrophilic carbon in the “in” complex and, particularly, on the Si face of the inner electrophilic carbon in the “out” complex. The intermediate is then C-protonated by neighboring His164 either directly or via an intermediate enol. In the pre-covalent complexes (Figure 8B, panels A and B), Cys88 and His164 are located on the same side with respect to the plane of the double bonds. Thus, the addition of the thiol and C-protonation of the enolate must occur in a syn fashion, as shown in Figure 8C, giving the corresponding covalent complexes (Figure 8C, right).

### 3.8. 2c Derivatives

Finally, we explored the possibility of modifying 2c (Table 1) with linkers for the future addition of smart moieties able to improve drug targeting/in vivo delivery, etc. 

Parent 2c was conjugated to different molecules via a carbamate linker. The latter was chosen because it allows for easy conjugation of the secondary alcohol group of 2c to primary amines in good yields (Figure S5). Furthermore, the carbamate group is chemically and metabolically stable and it is known to increase permeability across cellular membranes [26]. The compounds synthesized in this work (Table 1) can be grouped into two categories. The first set includes the parent compound 2c (1), alkyl and aryl carbamates (2–6), and derivatives (7–9) that are expected to be ionized at physiological pH. The second set includes compounds in which the parent 2c is linked to an amino acid. Phe and Leu were chosen because the hydrophobic side chains might interact favorably with hydrophobic regions near the isopeptidases’ binding site (vide infra). The amino acid was linked to the scaffold either directly, as in carbamates 10–12, or via a 1,3-diaminopropane linker, as in compounds **13**–**15**. Two compounds (**16**, **17**) in which the amino acid is directly linked to 2c by an ester bond were also synthesized for comparison. Compound 18, in which the parent 2c is conjugated to monomethoxy-PEG 5000 (MPEG5000) via a peptide linker, was also included in the investigation [23]. The addition of PEG 5000 confers hydrophilicity to 2c, allowing systemic administration (future tests) without using DMSO.

The activity of 2c derivatives was tested by calculating *IC*_50_ (Table 1). Only 11% and 16% of the derivatives were inactive in OVCAR3 and Kuramochi, respectively, indicating a remarkable adaptability of 2c concerning chemical modifications. Linear substituents are generally well tolerated (2, 3, 7, 8); however, a negatively charged substituent (at physiological pH) as in 9 significantly impairs the activity. This may be due to unfavorable interactions with the molecular targets and reduced cellular uptake. Extended aromatic substituents (4–6) do not improve the activity and have a variable effect, indicating that the orientation of the substituent may be important in the interaction with the target. The presence of amino acids, either attached directly to the scaffold (11, 12, 16, 17) or via a linker (13–15), is generally well tolerated; however, a cell-dependent effect takes place with compounds **13** and **14** being inactive in Kuramochi cells, while displaying a stereochemistry-dependent activity in OVCAR3. A negatively charged substituent (10) completely impairs the activity, as already in part observed for compound **9**. Finally, derivative 18, bearing a long linear chain linked to a hydrophilic, high-molecular-weight PEG molecule, shows an appreciable activity in both cell lines without the need for DMSO-mediated delivery.

## 4. Discussion

In the last 40 years, partial progress has been made concerning improvements in OC patient survival. Thus, novel therapeutic options are of utmost urgency. In this work, we have continued our investigation into the study of the effectiveness of the 2c molecule in OC. We have previously observed that 2c effectively induces cell death in several tumor cells, including glioblastoma, melanoma, lung/colorectal/hepatocellular/pancreatic cancer cell lines [6]. More recently, we observed that 2c may affect the viability of OC cancer cell lines, most likely by affecting the depolymerization of the F-actin network [11]. Here we have continued our investigation of 2c mechanisms of action, extending the exploration to patient-derived cancer cells (cultured in 2D/3D models) and to a xenograft mouse model of OC.

In 2D culture, 2c impairs OVCAR3/Kuramochi cell viability in a time- and dose-dependent manner (Figure 2). The effect is not identical between the two cell lines (see Table 1), a fact consistent with our previous observation that showed differences among cancer cell lines [6]. Despite this, the fact that Kuramochi cells have the highest genetic similarity to HGSOC compared to all the available OC cell lines, suggests the relevance of our findings for future clinical applications [12]. This concept is strengthened by the fact that 2c can effectively reduce the viability of primary patient-derived OC cells when cultured both in 2D (Figure 6A) and in 3D (PDOs, Figure 6B). Notably, 2c displays a significant broad potential in primary cancer cells derived from different OC types, including HGSOC (4 patients plus 2 from which PDOs were generated), clear cell OC (3 patients), serous ovarian cystadenoma (2 patients), and benign Brenner tumor (2 patients). Of note, 2c has a mild effect on cell viability in primary non-tumor ovary cells (Figure 6A, right). This opens the possibility of limiting the side effects of 2c in vivo, thereby alleviating one of the serious problems of current pharmacological treatments [27]. Despite this promising observation, we believe that 2c needs to be delivered conjugated with smart OC cell targeting moieties in the quest for future clinical application, aiming to improve treatment specificity [28]. In this regard, here we have started an investigation (see below).

2c can inhibit several cysteine-dependent DUBs, including UCHL5, altering protein degradation via the UPS [6]. We prove in the cellular models considered the expression of the deubiquitinase UCHL5 (Figure 7C), thus supporting the rationale for the study of 2c effects in OVCAR3/Kuramochi. Interestingly, 2c does not alter UCHL5 mRNA level (Figure 7C), reinforcing the concept that 2c affects UCLH5 at the post-translational level via docking to the enzyme active site (Figure 8). Notably, 2c affects proteasome enzymatic activities (Figure 7A,B): This most likely depends on the inhibition of UCHL5 that prevents protein deubiquitylation, a prerequisite for protein translocation to the proteasome catalytic core and thus to protein degradation [29]. Notably, 2c does not affect autophagy (Figure 3AIV,BIV). This is in part surprising, as in normal cells, autophagy activation represents a compensation mechanism for mediating the degradation of ubiquitinated proteins when the proteasome pathway is impaired [30]. A possibility is that in our tumor models, the compensatory role of autophagy is lost for reasons yet to be defined. Alternatively, we cannot exclude that 2c interferes with the autophagy pathway with unknown mechanisms that deserve future investigations.

The accumulation of polyubiquitinated proteins following DUB inhibition favors cell apoptosis, which we observed in OVCAR3/Kuramochi following 2c administration (Figure 3). However, compared to OVCAR3, in Kuramochi, apoptosis induction seems to be less pronounced, reflecting an increased resistance to the pro-apoptotic effect of 2c. The reasons for this observation deserve further investigation. However, Kuramochi seems to be more prone to necrosis induction at a high 2c dosage (10 μM) compared to OVCAR3 (compare in Figure 3 panels AIII vs. BIII). This may contribute to explaining the more evident reduction in Kuramochi cell number compared to the OVCAR3 one at 10 μM 2c (Figure 2C,D).

Our data indicate that the reduction in OC cell viability is not only due to increased apoptosis; it also depends on the impairment of the cell cycle (Figure 4A). This observation highlights a novel mechanism of action for 2c. Noteworthy, we observe an increase in G1/G0 cells, in line with the reduction in the protein level of E2F1 (Figure 4B,C) a known promoter of the G1 to S phase transition [24]. Notably, E2F1 overexpression has been associated with unfavorable disease-free and overall survival in OC [31]. Moreover, E2F1 expression is significantly increased in the more aggressive HGSOC compared to the less aggressive forms of OC [32]. The above consideration underlines the potential therapeutic significance of its expression inhibition by 2c. Whereas the mechanism by which 2c reduces E2F1 levels needs further investigation, we prove that E2F1 is functionally involved in the 2c anti-proliferative effect since its silencing reduces cell viability (Figure 4D,E).

Besides being effective in the 2D/3D cellular model of OC, we show that 2c is also effective in vivo in a mouse xenograft subcutaneous model of OC (Figure 5A). Concerning tumor growth, a mathematical analysis (Figure 5B) allowed us to determine the proliferation rate (VD^+^), which was significantly reduced in animals treated with 2c compared to control VV1-treated animals. In addition, this analysis demonstrated that the proliferation rate of 2c-treated animals becomes negative after ten days. Without this analysis, the effects of 2c could only have been roughly inferred from the growth curves. Thus, our mathematical model provides a powerful tool to accurately and objectively compare the efficacy of different anti-tumor approaches (different anti-tumor drugs, dosages, delivery systems, etc.) based on the tumor growth curve. This will be particularly useful in comparing the in vivo effectiveness of the derivatives developed here (future experiments).

Despite being effective in down-modulating OC cell survival in the models tested here, it is unlikely that 2c can be delivered in future clinical applications as done in the present work. Indeed, due to its hydrophobic nature, delivering it as a DMSO solution was necessary. A more hydrophilic form of 2c would be advantageous for delivery to aqueous environments such as blood (systemic delivery) or tissue (local delivery). The hydroxyl group on the cyclohexanone scaffold of 2c (Figure 1A) offers an ideal handle for the conjugation of small molecules and smart moieties able to confer optimal features such as hydrophilicity and the ability of OC cells to target. This group is distant from the electrophilic double bonds, and thus modifications at this site are not expected to affect their ability to react with biological thiols significantly. On the other hand, the docking studies indicate that, both in the non-covalent and covalent complexes with the representative deubiquitinase UCHL5 (Figure 8A,B), the OH group of 2c points towards the outside of the catalytic crevice, allowing some space for substituents grafted onto this functional group. Data in Table 1 indeed confirm that the biological activity is maintained for neutral and positively charged polar, hydrophilic compounds (**3**, **7**, **8**, **11**, **16**–**17**). Some differences are found between OVCAR3 and Kuramochi cells, notably for compounds **13** and **14** that are inactive in the latter model, indicating that the conjugation may affect drug trafficking within the cell.

Notably, we can reasonably exclude that differences in the activities of the 2c derivatives depend on the uptake; indeed, while we observed similar uptake between compounds **10** and **11** (Figure S1), the two derivatives resulted in no or excellent activity, respectively (Table 1). This suggests that the differences in activities are probably not related to cellular internalization but rather to other variables such as the compound’s ability to interact with the target enzyme.

Concerning the smart moieties bound to the linker, here we tested PEG (Table 1, compound **18**). PEG is a non-toxic and water-soluble polymer often used for drug delivery also in OC [28]. Our data show that compound **18** retains satisfactory effectiveness in both cellular models of OC. The PEG molecule is of considerable dimension, and it is likely to interfere with the binding of this conjugate to the narrow DUB target site. Compound **18** maintains good activity, which suggests that, upon entry into the cells, specific enzymes can cleave the linker at one of the peptide bonds, thus releasing 2c from the PEG carrier [6]. Further investigations are required to fully elucidate this aspect.

## 5. Conclusions

This work shows that 2c is effective in down-regulating the viability of various OC cell lines. This ability is maintained in primary tumor cells isolated from patients and cultured in 2D. The effectiveness is also confirmed when primary tumor cells are grown as organoids (PDOs), representing a valuable in vitro 3D model resembling different in vivo features of OC. The effectiveness is also observable in a subcutaneous xenograft mouse model of OC, in vivo. From a molecular perspective, we show that 2c triggers apoptosis most likely by interacting with DUB-UCHL5 via an appropriate docking (in silico evaluation) to the DUB active site. 2c also reduces cell growth by down-regulating the level of the transcription factor E2F1. It also induces cell necrosis and inhibits proteasome enzymatic activity. Finally, we show that 2c activity is often retained after conjugation with linkers usable to bind smart moieties for enhanced drug delivery/targeting/specificity. Collectively, our data strongly support the potential therapeutic value of 2c and its derivatives for the in vivo treatment of OC.

## Figures and Tables

**Table 1:** IC_50_ of 2c derivatives in OVCAR3 (OV) and Kuramochi (Ku).

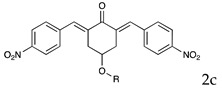			
Compound	Derivatization	IC_50_ μM OV/Ku	% Activity vs. 2c OV/Ku
**1**	H	6.22 ± 0.36/5.9 ± 0.41	100/100
**2**	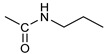	5.26 ± 1.71/7.89 ± 1.66	118/75
**3**	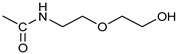	6.47 ± 0.83/5.91 ± 0.69	96/100
**4**	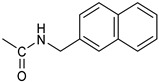	13.98 ± 3.37/5.21 ± 1.9	44/113
**5**	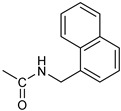	19.49 ± 0.67/7.8 ± 0.42	32/76
**6**	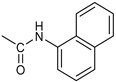	0/12.31 ± 2.07	0/48
**7**	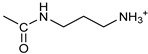	6.45 ± 1.04/10 ± 0.39	96/59
**8**	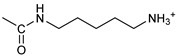	10.45 ± 0.69/7.95 ± 3.8	60/74
**9**	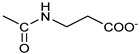	12.4 ± 0.76/50.7 ± 9.27	50/12
**10**	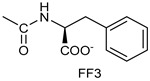	0/0	0/0
**11**	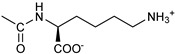	7.5 ± 0.4/6.1 ± 0.2	83/97
**12**	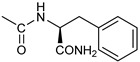	5.5 ± 1.8/6.7 ± 2.3	113/88
**13**	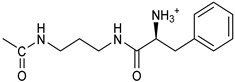	6.43 ± 0.32/0	97/0
**14**	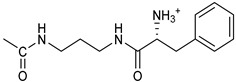	14.82 ± 0.74/0	42/0
**15**	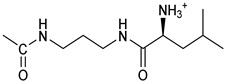	6.02 ± 1/9.51 ± 0.99	103/62
**16**	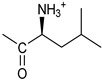	9.82 ± 1.62/11.4 ± 0.97	63/52
**17**	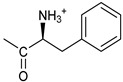	9.31 ± 0.6/11.47 ± 0.28	67/51
**18**	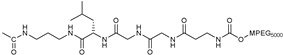	15.2 ± 1.2/8.7 ± 1.29	41/68

## Data Availability

The data presented in this paper are available upon request.

## Supplementary Materials

The following supporting information can be downloaded at: https://www.mdpi.com/article/10.3390/pharmaceutics16050664/s1, Figure S1: representative imagines of VV1 and compounds **10**/**11** uptake; Figure S2: Representative imagines of the morphology of OVCAR3 and Kuramochi cell lines following 2c administration; Figure S3: Trilencer siRNA uptake in OVCAR3 and Kuramochi; Figure S4: In silico docking of 2c to deubiquitinase UCHL5: molecular details; Figure S5: Conjugation of the secondary alcohol group of 2c to primary amines and to carboxylic acids.
