# Supplementary material for: In Vitro and In Vivo Evaluation of the Effects of Drug 2c and Derivatives on Ovarian Cancer Cells
Source: Pharmaceutics. 2024 May 15;16(5):664. doi: 10.3390/pharmaceutics16050664 (PMC11125437; doi:10.3390/pharmaceutics16050664)
Supplement: Supplementary file 1 [file pharmaceutics-16-00664-s001.zip › pharmaceutics-2985746-supplementary.pdf]

Figure S1. Representative imagines of the uptake of 2c derivatives n10/11 and VV1 fluorescently labelled in Kuramochi and OVCAR3 cell lines

A) Kuramochi

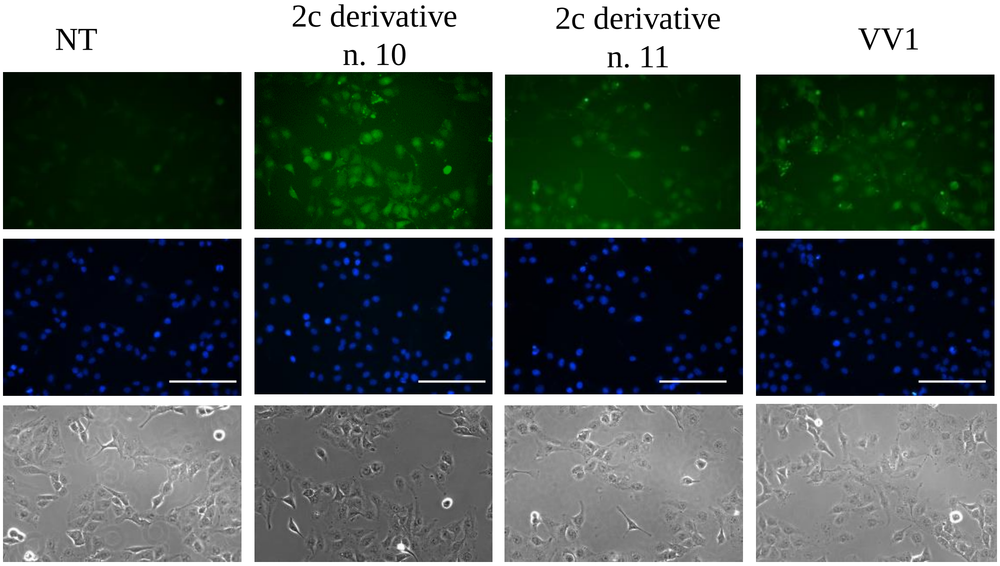

B) OVCAR3

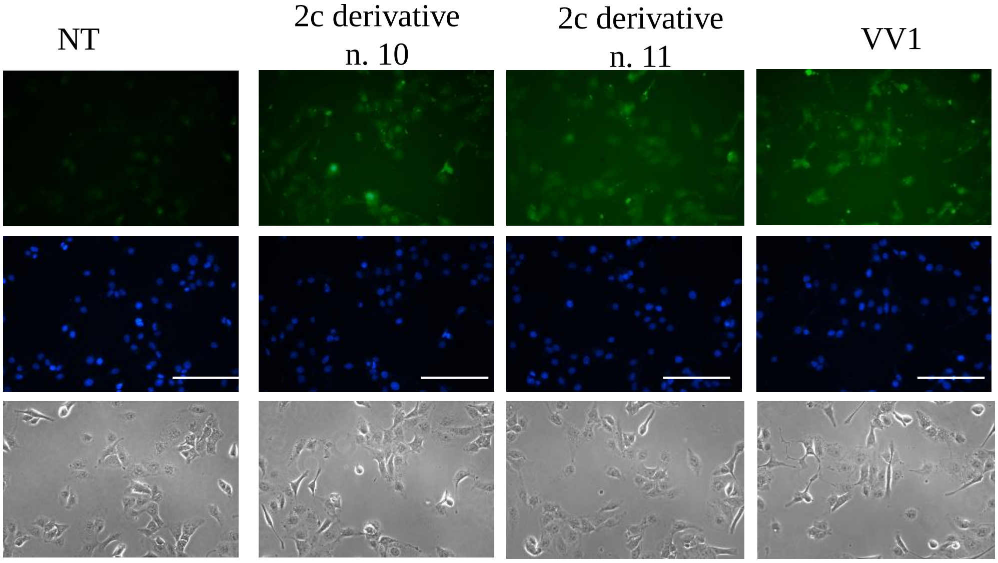

A) and B) Representative images of Kuramochi and OVCAR3, respectively, treated by the 2c-derivatives n10, n11 (see table 1) or VV1 (2 μM) conjugated with fluorescein; images were taken 24 hours after drug administration; scale bar: 100 μm, magnification 20x; nuclei have been labelled by DAPI (blue); images were taken with a Leica DM2000 fluorescence microscope.

Figure S2. Representative imagines of the morphology of OVCAR3 and Kuramochi cell lines following 2c administration

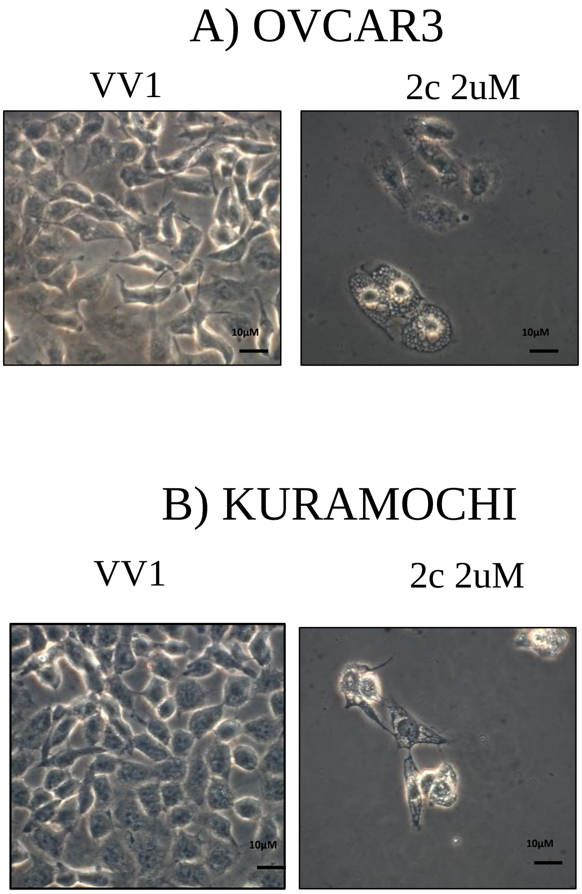

Representative imagines of the morphology of OVCAR3 (A) and Kuramochi (B) cell lines, analysed with a Leica DM 2000 phase contrast microscope, 24 hours after treatment with the 2c and the VV1 control, at a concentration of 2µM. (40/63x objective, Bar: 10µm).

Figure S3. Trilencer siRNA uptake in OVCAR3 and Kuramochi

OVCAR3

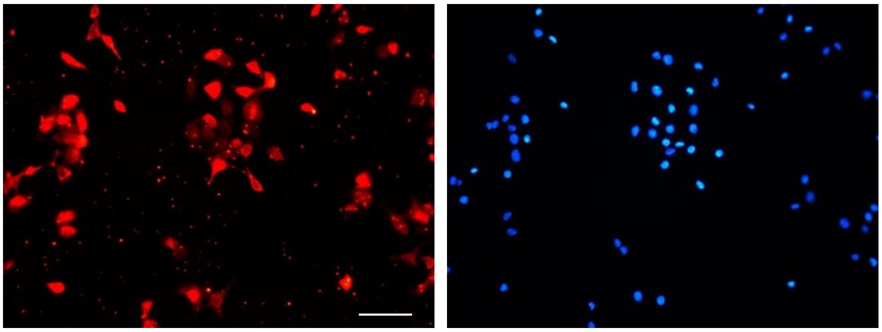

Kuramochi

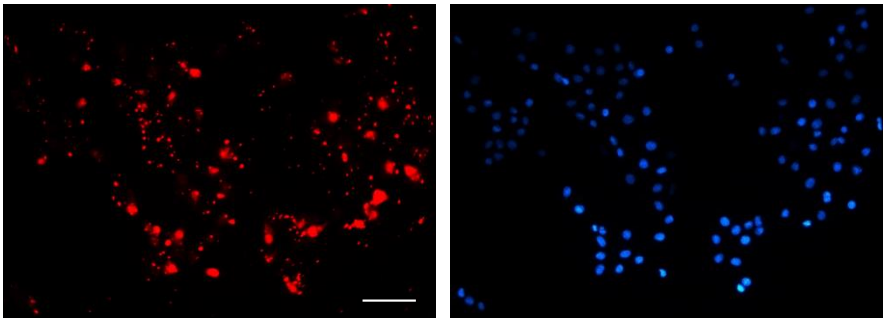

OVCAR3/Kuramochi were seeded at the density of 7,2*10^4 cells/well in six well plate on collagen I coated slide; transfection was carried 24 hours later for 4 hours using the red fluorescent Trilencer siRNA complexed with lipofectamine 2000. Imagines were taken immdediately after the end of transfection. Scale bar: 100 μm, magnification 20x; nuclei have been labelled by DAPI (blue); images were taken with a Leica DM2000 fluorescence microscope

Figure S4. *In silico docking of 2c to deubiquitinase UCHL5: molecular details*

A) Ligand interaction map for the “in” complex. Green: hydrophobic; cyan: polar; red: negatively charged.

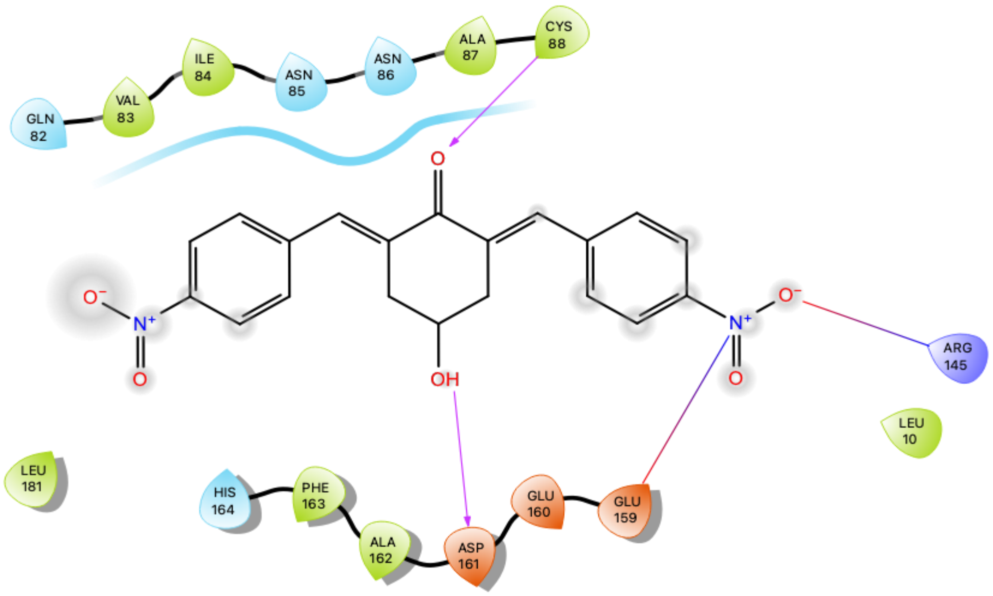

B) Ligand interaction map for the “out” complex. Green: hydrophobic; cyan: polar; red: negatively charged; violet: positively charged.

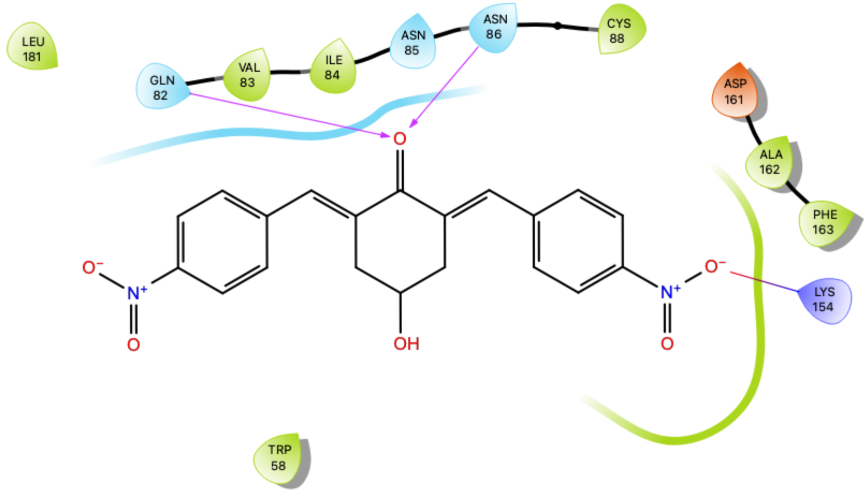

Figure S5. *Conjugation of the secondary alcohol group of 2c to primary amines and to carboxylic acids*

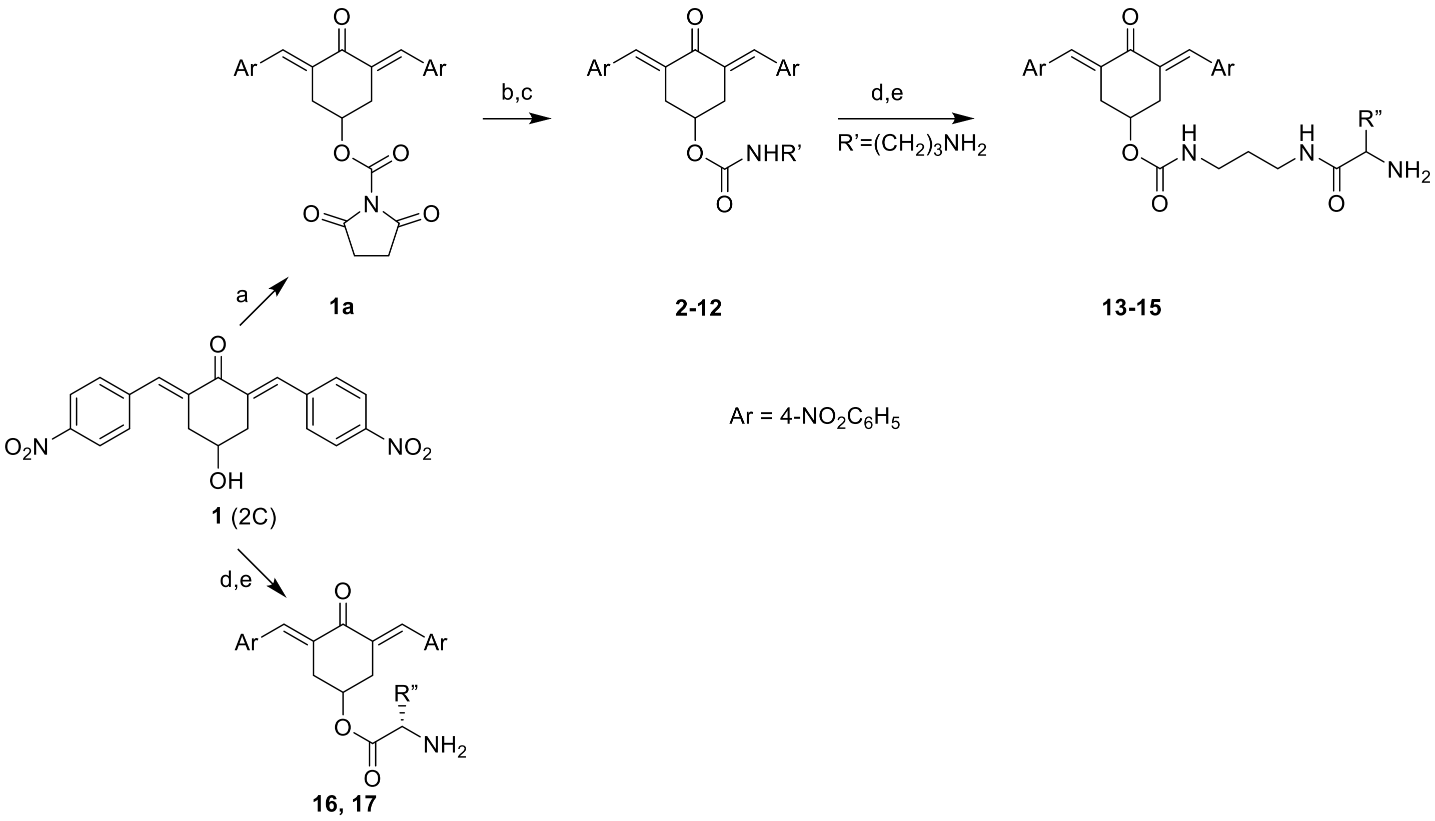

Reagents and conditions: a) N,N'-disuccinidimyl carbonate; b) R-NH_2, CH_2Cl_2, TEA, rt 12 h, 52-85%. c) for R=$(CH_2)_3$NHBoc and R=$(CH_2)_5$NHBoc: 10% TFA in DCM, rt, 12h; 82-85% overall. d) (L)-BocNHPhe or (L)-BocNHLeu, EDC, HOBt, CH_2Cl_2, TEA, 25 °C, 12h. e) 10% TFA in CH_2Cl_2, 25 °C, 12h, 62-70% overall.
